# The Microbial Composition in Circumneutral Thermal Springs from Chignahuapan, Puebla, Mexico Reveals the Presence of Particular Sulfur-Oxidizing Bacterial and Viral Communities

**DOI:** 10.3390/microorganisms8111677

**Published:** 2020-10-29

**Authors:** Hugo Gildardo Castelán-Sánchez, Pablo M. Meza-Rodríguez, Erika Carrillo, David I. Ríos-Vázquez, Arturo Liñan-Torres, Ramón Alberto Batista-García, Ernesto Pérez-Rueda, Norma Elena Rojas-Ruíz, Sonia Dávila-Ramos

**Affiliations:** 1 Centro de Investigación en Dinámica Celular, Instituto de Investigación en Ciencias Básicas y Aplicadas, Universidad Autónoma del Estado de Morelos, Cuernavaca 62209, Morelos, Mexico; pablo.manuel.mz@gmail.com (P.M.M.-R.); cerika996@gmail.com (E.C.); arturo21lt@gmail.com (A.L.-T.); rabg@uaem.mx (R.A.B.-G.); 2 Centro de Investigaciones en Ciencias Microbiológicas, del Instituto de Ciencias, Benemérita Universidad Autónoma de Puebla, Puebla 72570, Mexico; david.rios.vazquez@gmail.com (D.I.R.-V.); normaelena_rojas@yahoo.com.mx (N.E.R.-R.); 3 Instituto de Investigaciones en Matemáticas Aplicadas y en Sistemas, Sede Mérida, Universidad Nacional Autónoma de México, Unidad Académica Yucatán, Mérida, Yucatán C.P. 97302, Mexico; ernesto.perez@iimas.unam.mx; 4 Centro de Genómica y Bioinformática, Facultad de Ciencias, Universidad Mayor, Providencia, Santiago C.P. 7500000, Chile

**Keywords:** thermophilic bacteria, AMG viral genes, terrestrial thermal spring

## Abstract

Terrestrial thermal springs are widely distributed globally, and these springs harbor a broad diversity of organisms of biotechnological interest. In Mexico, few studies exploring this kind of environment have been described. In this work, we explore the microbial community in Chignahuapan hot springs, which provides clues to understand these ecosystems’ diversity. We assessed the diversity of the microorganism communities in a hot spring environment with a metagenomic shotgun approach. Besides identifying similarities and differences with other ecosystems, we achieved a systematic comparison against 11 metagenomic samples from diverse localities. The Chignahuapan hot springs show a particular prevalence of sulfur-oxidizing bacteria from the genera *Rhodococcus*, *Thermomonas*, *Thiomonas*, *Acinetobacter*, *Sulfurovum*, and *Bacillus*, highlighting those that are different from other recovered bacterial populations in circumneutral hot springs environments around the world. The co-occurrence analysis of the bacteria and viruses in these environments revealed that within the *Rhodococcus*, *Thiomonas*, *Thermonas*, and *Bacillus* genera, the Chignahuapan samples have specific species of bacteria with a particular abundance, such as *Rhodococcus erytropholis*. The viruses in the circumneutral hot springs present bacteriophages within the order Caudovirales (Siphoviridae, Myoviridae, and Podoviridae), but the family of Herelleviridae was the most abundant in Chignahuapan samples. Furthermore, viral auxiliary metabolic genes were identified, many of which contribute mainly to the metabolism of cofactors and vitamins as well as carbohydrate metabolism. Nevertheless, the viruses and bacteria present in the circumneutral environments contribute to the sulfur cycle. This work represents an exhaustive characterization of a community structure in samples collected from hot springs in Mexico and opens opportunities to identify organisms of biotechnological interest.

## 1. Introduction

Terrestrial thermal springs are widely distributed throughout the world. They harbor a significant number of microorganisms of biotechnological interest. These ecosystems have been classified in low-temperature (<55 °C) and high-temperature (>55 °C) springs; in terms of pH, the springs are acidic (pH < 4), intermediate (pH ~4), circumneutral or neutral (pH ~7), or alkaline (pH > 7) [1,2,3]. Additionally, the thermal springs are classified according to their origin in magmatic waters, which are born in volcanic areas and at high temperatures (>50 °C), and telluric waters, which are formed when underground water currents pass along deep hot rocks [4].

The most studied thermophilic environment in the world is Yellowstone National Park (YNP) (pH 2, 75 °C) [1,5], where pioneering studies opened the possibility of exploring the diversity of microorganisms in extreme environments, as well as the genes that encode enzymes with biotechnological applications [3,6,7,8]. Recently, a growing interest in studying these ecosystems has emerged [1,9,10,11,12,13,14,15,16,17,18,19,20,21,22], and these studies have focused on the sites of high temperatures and acidic or alkaline pH [15,16,19,23,24,25], and have identified a high diversity of microorganisms [22,26]. Intermediate or circumneutral hot springs also exhibit a high diversity of microorganisms; biodiversity generally decreases with increasing temperature and decreasing pH [21]. 

Mexico contains a wide diversity of thermal springs, steam vents, geothermally heated soils, boiling mud pools, and geothermal zones [25,27,28]; however, few studies have described the diversity of microbial communities in thermal environments. In particular, in the acidic hot spring “*Los Azufres*” (pH 3.6 and 65 °C) located in the state of Michoacan, *Rhodobacter*, *Acidithiobacillus*, and *Lysobacter* [25], among other bacteria, have been identified; whereas the *Sulfolobales archaeon* has been discovered in “*Los Azufres*” [29]. Finally, the viruses identified correspond to archaeal *Fusellovirus*, archaeal *Rudivirus*, and *Sulfolobales Archaeon AZ1* [30]. In addition, in a hot spring located in the Araro region, Michoacan, different genera of bacteria were found, such as *Bacillus*, *Aeromonas*, and *Pseudomonas* [31,32], whereas in the “*Carrizal*” thermal pool hot spring and in “*Los Baños*” in Veracruz, Mexico, bacteria of the genera *Geobacillus*, *Anoxybacillus*, and *Aeribacillus* have been identified [33]. 

In this work, we explore the microbial community and functional composition in the thermal spring “*Baños Termales de Chignahuapan*”, which is located in the geothermal region of Tulancingo-Acoculco, Sierra Norte, Puebla. This is a mountainous complex, whose origin dates back to the Pleistocene (1.7–0.9 million years ago), where there is a magmatic hot spring with travertine sediment compositions and rocks of dacites and rhyolites [27,28]. 

We consider that the hot springs in Chignahuapan present a particular combination of physicochemical characteristics, such as high concentrations of calcium, carbonate, and sulfur, making it an excellent spot to determine the microorganisms and viruses that make up that ecosystem, as well as their functional potential. In addition, we achieved a comparative analysis with 11 circumneutral hot springs, to determine differences in composition and diversity in microorganisms and highlight the influence of the environment in the community structure.

## 2. Materials and Methods

### 2.1. Physicochemical Characterization

The temperature of thermal water was measured in situ. A 1 liter water sample was analyzed in the laboratory to determine the physicochemical parameters. The pH was determined by the method established in NMX-AA-008-SCFI-2016 [34]; electrical conductivity reported in deci-siemens (dS) was measured on a HANNA conductivity meter; the ion’s calcium (Ca^2+^ mg L^−1^) and magnesium (Mg^2+^ mg L^−1^) were determined by the EDTA method; sodium (Na^+^ mg L^−1^) and potassium (K^+^ mg L^−1^) were evaluated with flamometry; nitrates (NO_3_ mg L^−1^) were determined by the methodology cited in NMX-AA-079-SCFI-2001 [35]. Sulfates (SO_4_^−2^ mg L^−1^) were determined by the NMX-AA-074-SCFI-2014 [36]; carbonates (CO_3_^−2^ mg L^−1^) were evaluated according to NMX-AA-029-SCFI-2001 [37], and bicarbonates (HCO_3_^−1^ mg L^−1^) were determined by volumetric methods; chlorides (Cl^−1^ mg L^−1^) were analyzed according to NMX-AA-073-SCFI-2001 [38].

### 2.2. Sample Collection and Processing from Chignahuapan Puebla, Mexico

Two samples of water (20 L) were collected from the recreational center “Baños Termales de Chignahuapan” located at coordinates 19°50’30’ N 97°59’41’’ W, at an altitude of 2136 m above sea level, during April 2019. The samples were obtained with sterilized tools in 1 L containers, from the water emerged in the mountain before the pools were supplied, and transported at room temperature to the laboratory of the Benemérita Universidad Autónoma de Puebla, where they were filtered through 0.22 µm Millipore filters. The DNA was obtained from the filters and isolated using ZymoBIOMICS DNA kits (MoBio, West Carlsbad, CA, USA). The DNA concentration was determined using a NanoDrop 1000 spectrophotometer (Thermo Scientific), and fluorometry was measured using a Qubit 4 fluorometer (Invitrogen). DNA was sequenced using the Illumina NextSeq 500 platform with the Nextera reagent kit V3.0 for a read length 2 × 75 bp at the Instituto de Biotecnología of Universidad Nacional Autónoma de México. 

### 2.3. Taxonomic Annotation of Metagenome

We analyzed two metagenomic samples from Chignahuapan, Puebla, and 11 shotgun metagenomic samples retrieved from the Sequence Read Archive (SRA) database, from thermal environments with physicochemical characteristics of circumneutral hot spring and pH 7 (Table S1). The quality control of sequences and the removal of adapters was performed by using Trimmomatic v.036 [39] with a sliding window of 4 bp, an average quality per base of 30, and a minimum read length of 75 bp. Reads were assembled in contigs using MEGAHIT [40], under default parameters in paired-end mode, and contigs with a minimum length of 1000 bp were considered for further analysis.

Taxonomic assignments were performed with the software Kaiju v1.7.3 [41] against the nonredundant protein database v1.7 sourced from the National Center for Biotechnology Information (NCBI) databases using the maximum exact matches, and 11 as a minimum match length. Finally, the results were displayed with the library Pavian in R [42]. 

The composition of the prokaryotic communities was evaluated using statistical analyses in R [43]. The nonmetric multidimensional scaling (NMDS) plot was performed in Vegan v2.3-1, with the stress function, to determine the goodness.

The taxonomic profiles at the genus level were used to calculate the diversity indices from all data. Diverse alpha-diversity descriptors were obtained using the Phyloseq function in R [44]. 

The beta diversity was determined by Bray–Curtis dissimilarity, and the sampling effort was evaluated through the rarefaction curves using the Vegan library implemented in R [45].

The metagenomes were deposited in the Joint Genome Institute (JGI) Integrated Microbial Genomes and Microbiomes database, with accession number: Gs014786.

### 2.4. Co-Occurrence Network Analysis 

The co-occurrence analysis was implemented using the igraph library [46] and bipartite library in R [47], implemented under development Virome Network Analysis (ViNA) [48]. In brief, we computed a table of incidences of the relevant bacteria and viruses at the species level. These tables indicate the presence or absence of each taxon in the metagenome. After that, the network displayed the taxon associations and locations, which was built using the Kamada–Kawai algorithm layout [49].

### 2.5. Identification and Annotation of Viral Genomes

For virus classification, two approaches were implemented. In the first approach, Virsorter [50] was used to determine the viral contigs, using the viral hallmark genes annotated as “main capsid protein”, “portal”, “large subunit of the terminase”, “tail”, and “envelope”, among others. The entire contig was considered viral if more than 80% of predicted genes on a contig had a viral signal. This software finds new viruses with different confidence categories from 1 to 6, with 3 and 6 as the least confidence level.

The categories 1, 2, 4, and 5 were concatenated, and contigs were compared with BLASTn against the nonredundant database (nr), with the following parameters: -num_alignments 20, -num_descriptions 20, e-value 0.0001, -word_size 11. The results were visualized in MEGAN v5.10.6 considering the lowest common ancestor (LCA) method with the following parameters that reduce the rate of false positives and false negatives [51]: minimum support of 2; minimum score of 70; top percent of 10.

In the second approach, the viral contigs were recovered and the auxiliary metabolic genes (AMG) were obtained using the program VIBRANT [52]. This program is a hybrid machine-learning algorithm and similarity comparisons of protein sequences. It annotates the genes supporting metabolism and recovers the metabolic pathways where these genes are involved. We considered a minimum length of 1000 bp, with summary plots on and function virome off. The retrieved contigs were compared with BLASTn against the Viral RefSeq database and analyzed using the software MEGAN v5.10.6 with the same conditions [51].

### 2.6. Functional Annotation 

To predict protein-coding genes in the assembled contigs, we used Prodigal v2.6.3 [53] with the metagenomic mode. The functional annotation was achieved using SUPERFOCUS [54], which contains the SEED database with an E-value of 0.0003 and 60% identity. The results were displayed in a heatmap using the ggplot2 library in R [55]. The metabolic pathways were displayed using MG-RAST server with the database KO [56].

## 3. Results and Discussion 

### 3.1. Field Sampling and Physicochemical Characterization

The water samples were collected from two thermal springs at Chignahuapan, Puebla, Mexico. The first sample was collected from the thermal spring (Mex_Chig_S1) with a temperature of 49–50 °C, and a pH of 7.02. The second sample was collected from the water that supplies the pool (Mex_Chig_S2). This sample temperature was 45 °C and the pH was 6.66. 

The compositions of both samples were compared with respect to the content of different salts, quantified in Table 1. The sulfate was found with a concentration of 25.6 and 30.2 mg L^−1^ in Mex_Chig_S1 and Mex_Chig_S2, respectively; in the site, there was an intense smell of hydrogen sulfide (H_2_S), indicating that the sulfate was being reduced to hydrogen sulfide by sulfate-reducing microorganisms. These concentrations were in the range of thermal spring waters. In contrast, when Ca^2+^, carbonate (HCO_3_^−1^), and Na^2+^ were quantified, these ions were present in high concentrations, suggesting that they are out of range according to water quality standards in Mexico.

The presence of high levels of carbonates and calcium in both samples collected could be associated with hydrothermal travertine deposits found in hot springs, as these deposits are mainly composed of CaCO_3_ (calcite) [6,19]. Likewise, there are previous reports of the presence of travertine deposits (calcium carbonate), rhyolite, and dacite in the Chignahuapan springs [28], and the presence of these carbonates could be involved in the modification of the microbial structures of the communities.

The structure of the microbial community is also modified by the concentration of Na^+^; it is known that decreased concentrations of salts lead to a higher diversity of bacteria, while archaea are abundant in a higher concentration of salts [57,58]. In the thermal environments of Puebla, a concentration outside the range for consumption according to the Mexican regulations was also found; thus, the high salt concentrations found in both samples will determine the diversity and structure of microbiomes in thermal springs. 

### 3.2. Microbial Community Composition of Chignahuapan Metagenomes

**Figure 1:**
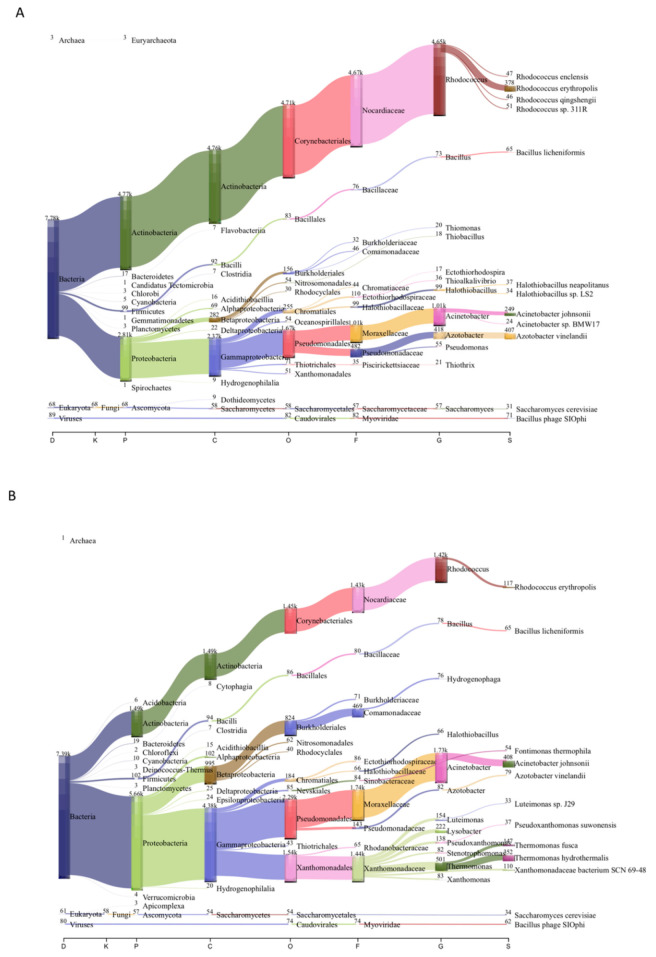
Taxonomic profile in circumneutral thermal spring metagenomes from Chignahuapan, Mexico. (**A**) Mex_Chig_S1. (**B**) Mex_Chig_S2. On the x-axis are the taxonomic levels: D, domain; P, phylum; C, class; O, order; F, family; G, genus; S, species. The numbers correspond to the assigned contigs.

The diversity and abundance of microorganisms in the circumneutral thermal spring samples from Chignahuapan, Puebla, were determined by shotgun metagenomic sequencing. From these metagenomes obtained from two locations, the diversity of the microbiome was determined. To this end, the assembled contigs were classified with Kaiju (Table 2), and the results at the domain level showed that Bacteria represent between 88.4 and 91.8% of the total microorganisms, followed by Archaea (1.3–1.8%), Viruses (0.8–0.9%), and Eukarya (0.7–0.8%) (Figure S1). The microbial composition in the metagenomes was in concordance with that found in similar environments with a neutral pH, moderate temperature (50 °C), and similar chemical composition, such as the Jordanian hot springs [17,21] (Figure 1). 

In the sample Mex_Chig_S1, bacteria from the phylum *Actinobacteria* (64.21%) were the most abundant, followed by Proteobacteria (36.09%). In contrast, in the sample Mex_Chig_S2, Proteobacteria was found as the most abundant (76.81%), followed by *Actinobacteria* (20.14%) (Figure 1). This result is consistent with previous descriptions in circumneutral water, sulfur water springs, and volcanic terrain, respectively, where Proteobacteria and Actinobacteria are predominant in sulfur water springs and volcanic terrain, respectively [17,48]. 

In Mex_Chig_S1, the most abundant genera were *Rhodococcus* (59%), followed by *Acinetobacter* (13%), *Azotobacter* (5%), *Halothiobacillus* (1.2%), and *Bacillus* (0.9%). At the species level, *Rhodococcus erythropolis* was identified as the most abundant (Figure 1). It is worth mentioning that *R. erythropolis*, originally isolated from crude oil [59], is a biologically important bacterium because it possesses selective desulfurization activity and the capacity to degrade alkanes (C_8_ to C_20_ n-alkanes) and methyl benzenes such as toluene [59,60]. 

Another interesting genus was *Halothiobacillus*, which is an obligate chemolithoautotroph and sulfur oxidizer. In particular, *Halothiobacillus neapolitanus* was found in the samples that encode a complete SOX complex, involved in sulfur oxidation [61].

At the species level, *Bacillus licheniformis* (0.8%) was also found—an interesting bacterium which was also isolated from Jordanian hot springs [17]. *Bacillus licheniformis* is widely distributed in thermal springs, and it is considered for commercial use as it has been used in the production of enzymes, antibiotics, and detergents, but some species of *Bacillus* are involved in carbon metabolism [62,63].

In Mex_Chig_S2, *Acinetobacter* (23%) was the most abundant genus, followed by *Rhodococcus* (19%), *Thermomonas* (6.8%), *Lysobacter* (3%), *Luteimonas* (2%), *Pseudoxanthomonas* (1.8%), and *Xanthomonas* (1.1%). 

At the species level, the most abundant were *Thermomonas hydrothermalis* (4.1%), *Thermomonas fusca* (1.7%), *Rhodococcus erythropolis* (1.3%), *Xanthomonadaceae bacterium* SCN 69-48 (1.3%), and in less than 1% of species such as *Fontimonas thermophila*, *Sulfurovum* sp. enrichment culture clone C5, *Thiomonas bhubaneswarensis*, *Thiomonas intermedia*, and *Sulfurovum* sp. AS07-7, were found; these bacteria are important because they are involved in the sulfur metabolism and the capability to oxidize sulfur [64] (Figure 1, Figure S2).

Overall, these bacteria are moderate thermophilic organisms isolated from hydrothermal springs with diverse enzymatic activities characterized as amylases, cellulases, and lectinases [65]. *T. bhubaneswarensis* and *T. intermedia* are widely distributed in hot springs from India, which are rich in arsenic and contain low levels of organic matter; these bacteria are sulfur and thiosulfate-oxidizing [66].

The sulfur-oxidizing bacteria were highly abundant in both samples, correlating with the concentrations of sulfate in water. These bacteria can oxidize sulfur compounds (thiosulfate, tetrathionate, sulfide, and polysulfide) to produce energy, which has been previously reported in high-temperature sulfidic hot springs [14]. 

In general, these bacteria belong to Actinobacteria, Gammaproteobacteria, Betaproteobacteria, and Epsilonproteobacteria phyla, chemoheterotrophs or chemolithoautotrophs in the microbial community, with the ability to use electrons from inorganic compounds as an energy source. Overall, many of them are sulfur-oxidizing bacteria.

In the circumneutral thermal spring samples from Chignahuapan, Puebla, archaeal organism abundance was low, similar to findings in other circumneutral hot springs [17,50]. This archaeal composition is similar to those found in Malaysia’s circumneutral hot spring, where a low proportion of archaea was found [67,68]. The most abundant classes identified in our samples correspond to Halobacteria within the Euryarchaeota phylum. Within the few species found were *Halolamina sediminis* and *Candidatus altiarchaeum* sp. Both are interesting because the first is associated with hypersaline aquatic environments, which may be possible due to the high salt concentrations present in Chignahuapan, and the second is involved with carbon fixation and plays an essential role in biogeochemical cycles [69,70]. These results contrast with previous reports indicating that Crenarchaeota is most abundant in terrestrial thermophilic environments in acid hot springs with high temperatures [63,65]. 

### 3.3. Comparative Analysis and Ecological Indices 

To determine whether the samples of circumneutral hot springs from Chignahuapan, Puebla, share similarities with other hot springs samples, we compared the microbial community with 11 circumneutral metagenomes from the SRA database. These were obtained from different sites in the world, selected based on physicochemical characteristics similar to those of the sample’s Mex_Chig_S1 and Mex_Chig_S2 (Table S1). The aquatic thermal terrestrial metagenomes were selected with a nearly neutral pH. 

**Figure 2:**
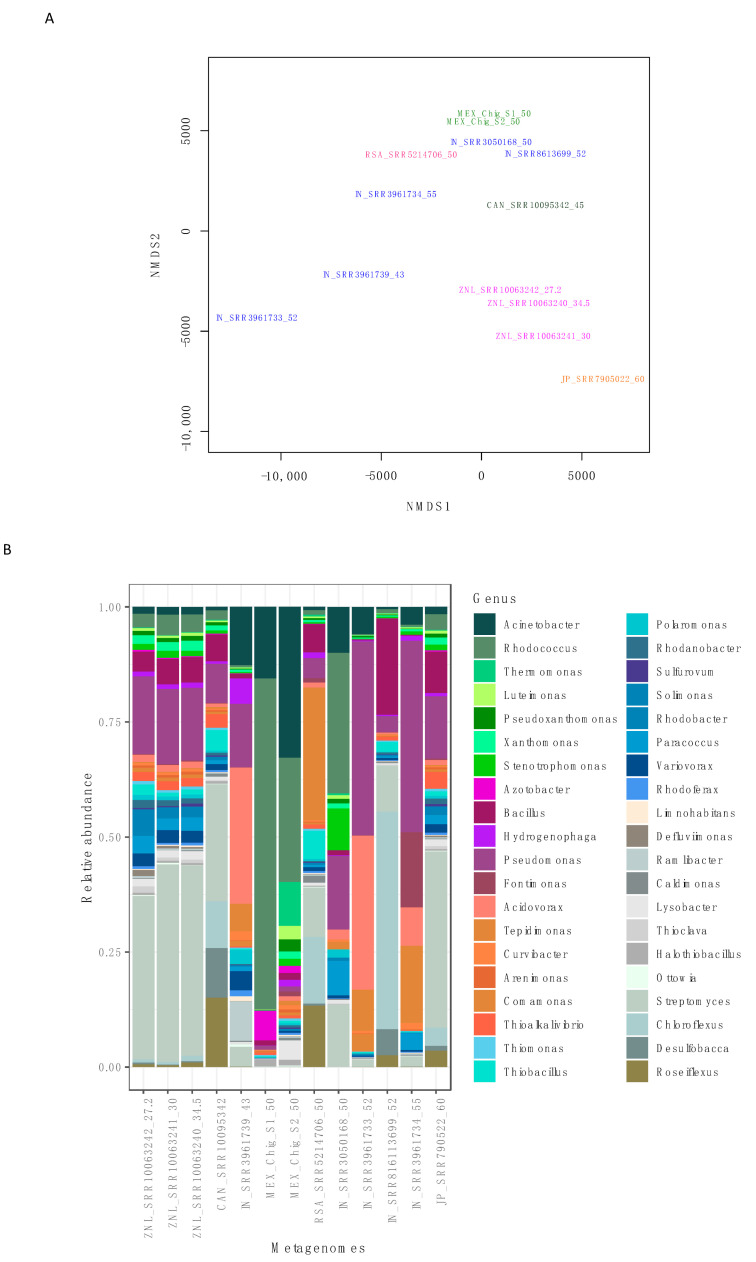
Comparison of hot spring metagenomes at the genera level. (**A**) nMDS analysis, using a Bray–Curtis distance between samples. On the x-axis is dimension 1 and on the y-axis is dimension 2. (**B**) Relative abundances of 30 bacterial genera of metagenomes.

The comparison showed that the Puebla hot springs metagenomes did not have a similar bacterial composition to the other metagenomes. To analyze this, we carried out a nonmetric multidimensional scaling (nMDS) analysis. The nMDS analysis showed that the Chignahuapan samples were grouped together, while the other metagenomes were grouped according to their geographical area, with the stress of 0.03; the lower the stress value, the better the goodness of fit (Figure 2A).

Likewise, differences were found in the composition at the genus level in the bacterial communities. The microbial community structure was particular in the Chignahuapan samples, mainly of the *Rhodococcus* genus, which was found in great abundance, followed by genera such as *Acinetobacter*, *Thermomonas*, and *Azotobacter* (Figure 2B).

This particular community of microorganisms is possibly associated with the physicochemical characteristics, including abiotic factors such as Ca, SO_4_, HCO_3_ ion levels, that have previously been reported to have an essential role in the microbial composition and have been associated with some bacteria, such as *Thermomonas hydrotermalis*, *Bacillus licheniformis*, *Bacillus subtilis*, and *Anoxybacillus kamchatkensis* [6]. The composition of the bacteria and minerals reported is similar to those of Chignahuapan; therefore, the water’s physicochemical factors very likely influence the structure of the microbial community.

Concerning the other metagenomes, the IN_SRR3050168_50 and IN_SRR8613699_52 from India were clustered near the Mexican samples, and the others, IN_SRR3961733_52, IN_SRR3961734_55, and IN_SRR3961739_43 samples were separated from all others (Figure 2A). In this regard, the genera *Acidovorax*, *Microbacterium*, *Pseudomonas*, and *Caulobacter* were associated with India’s location. A similar finding was observed with the samples from New Zealand, ZNL_SRR10063242_27.2, ZNL_SRR10063241_30, and ZNL_SRR10063240_34.5, where *Streptomyces*, *Phenylobacterium*, and *Asticcacaulis* were clustered. The hot springs of Canada (CAN_SRR10095342_45) and Japan (JP_SRR7905022_60) were clustered together, whereas the genera, *Streptomyces*, *Roseiflexus*, *Desulfobacca*, and *Chloroflexus*, were clustered (Figure 2B and Figure S3). 

The results show differences in the recovered genera and that they are grouped by geographic area. However, this grouping can also be driven by the composition of ions, minerals, and elements present in the water. In previous studies, measurements of different elements, compounds, and ions were taken in the water samples. For example, in India’s selected samples, which have high concentrations of Co, La, Fe, Hg, and Si, the predominant bacteria were *Pseudomonas stutzeri* and *Acidovorax* sp.; these findings are in accordance with the taxonomic assignment made by us [9]. Furthermore, samples from another study from the same country were nearly clustered with them. However, in this other site, different dissolved solids were measured: there were high concentrations of phosphorus and sulfur (Figure S3), and the genera *Microbacterium*, *Propionibacterium*, *Caulobacter*, and *Rhodococcus* predominated, among others [71]. 

Whereas the New Zealand samples had different values in the parameters evaluated, one of the samples had a high concentration of methane (ZNL_SRR10063242_27.2), and ammonia (ZNL_SRR10063240_34.5) and iron were present in all the samples. The genera that predominated in these metagenomes were different than those found in the other metagenomes [72].

On the other hand, in the Japanese metagenome (JP_SRR7905022_60), high levels of iron and dissolved oxygen have been reported, and these findings correlate with the more abundant bacteria genus *Chloroflexus*, which has photosynthetic activity [73]. 

However, the differences observed in the microbial community are associated with the temperature presented by thermal environments. For example, a study of the hot springs in Canada and New Zealand showed that some phyla had trends that changed with temperature, where Cyanobacteria, Acidobacteria, Verrucomicrobia, and Planctomycetes were absent at high temperatures, while other phyla did not show changes [74]. In our cluster analysis, we can also observe that there is certain proximity of the metagenomes that share a similar temperature (Figure 2). 

The multivariate approaches performed revealed that diversity patterns changed in each geographical location, where specific genera predominated in each of the metagenomes, and possibly this predominance was a consequence of the different physicochemical compositions and temperatures present in the water [26]. 

These differences can also be clearly distinguished in the relative abundance analysis. Proteobacteria were predominant, correlating with other moderate temperature circumneutral springs, such as the Jordanian Main springs, where about 50% corresponds to these phyla [17]. However, Puebla’s microbial community structure was mainly compared to other terrestrial hot springs (Figure S3). Overall, these results indicate that the microorganism communities change depending on the geographical area and physicochemical composition (Figure S3). 

**Figure 3:**
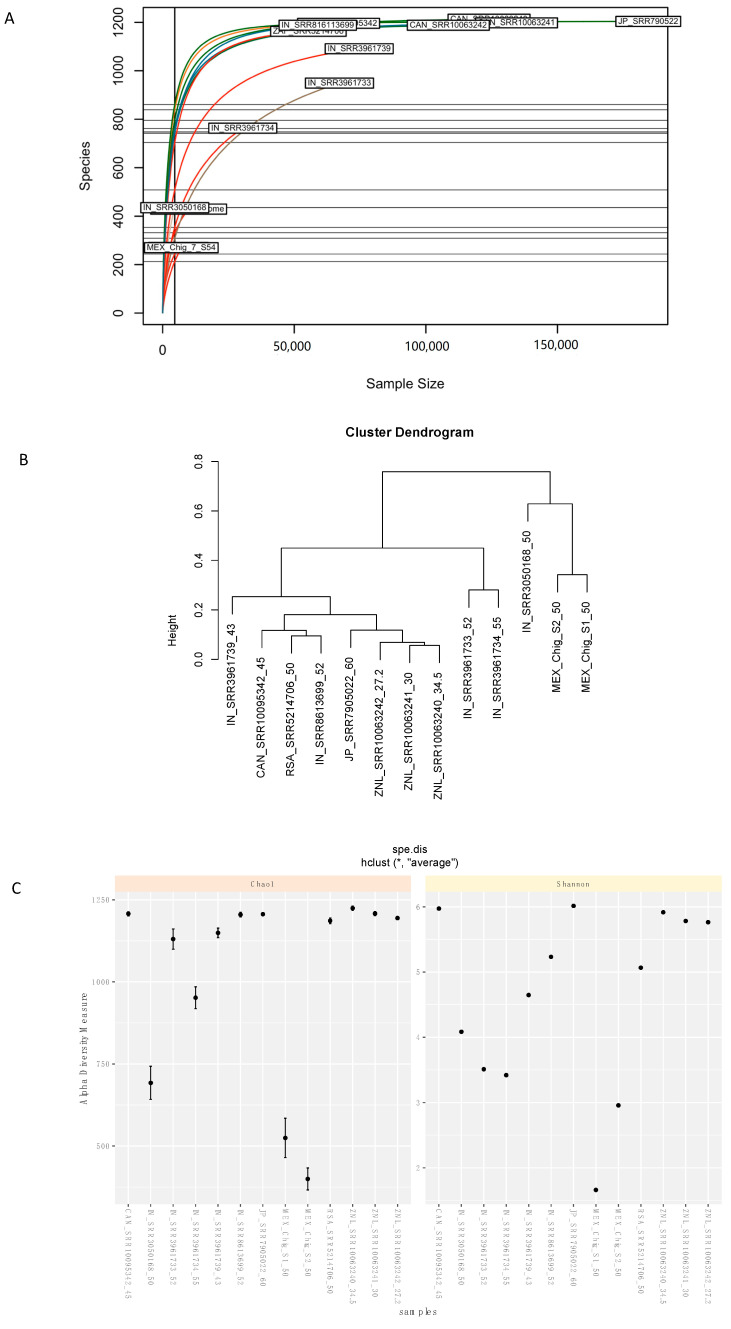
Analysis of diversity and enrichment of species. (**A**) Species-accumulation curves at the genus level. Rarefaction curves of the 13 hot spring metagenomes; the curves from Chignahuapan do not have asymptotic behavior compared to the others. (**B**) Box plots of alpha diversity. (Chao1 and Shannon). (**C**) Beta diversity dendrogram, showing dissimilarity between the metagenomes, on the x-axis is the metagenomes, and on the y-axis is alpha diversity.

The circumneutral hot springs were evaluated through the ecological indices or diversity indices, and species accumulation curves were compared among all the metagenomes. The rarefaction curve associated with the Chignahuapan metagenomes showed a low number of species, probably due to a low yield obtained from the sequencing run; therefore, these samples did not have asymptotic behavior (Figure 3A). When the alpha and beta biodiversity indices were analyzed, a variation was shown along with the α-index. 

Alpha-diversity values were lower for the Chignahuapan samples compared to the other metagenomes. However, it has been reported that diversity decreases with increasing temperature; these studies showed that the alpha-index is higher in those places with a neutral pH and a temperature of 50 °C [74]. Therefore, it was expected that the samples from Mexico had high diversity indices compared to other samples. In the case of the samples analyzed, the sample from Japan had the highest alpha index, compared to the other metagenomes. Therefore, it can be suggested that while diversity is modified by physicochemical changes, it is also modified by experimental yields and conditions.

The low values obtained for the β-diversity suggest that Chignahuapan metagenomes are less diverse than the other metagenomes (Figure 3B). In this regard, the clustering using β-diversity showed that the hot spring metagenomes were clustered primarily based on geographic locations and temperature. The Chignahuapan thermal springs clustered independently, indicating that they have a different microorganism community than the other metagenomes (Figure 3C). Interestingly, in this analysis, the sample from India was grouped with the samples from Mexico. The Indian sample also had low diversity indices indicating less diversity.

In summary, comparative analysis shows that Chignahuapan metagenomes are less diverse than other hydrothermal environments. However, they have a unique microorganism composition, because they do not group with the other samples with similar temperatures. Therefore, the chemical composition of water could determine the microbial structure of Chignahuapan hot springs. 

### 3.4. Co-Occurrence Network Analysis 

We performed a co-occurrence network analysis to evaluate possible genera interactions between the 13 metagenomes. Each of them evaluated the association between species and metagenomes; the genus *Rhodococcus* (Actinobacteria) was considered because it is the most abundant bacterial genus in Chignahuapan samples. Another of the abundant phyla was Proteobacteria. However, two genera of sulfur-oxidizing Beta-proteobacterium were considered in this analysis (*Thiomonas* and *Thiobacillus*) and *Bacillus* (Firmicutes). Although these genera were not the most abundant, they are sulfur-oxidizing bacteria, and so it is interesting to observe their interaction; the main network metrics were evaluated, which were connectance, nestedness, modularity index, and weighted closeness. 

**Figure 4:**
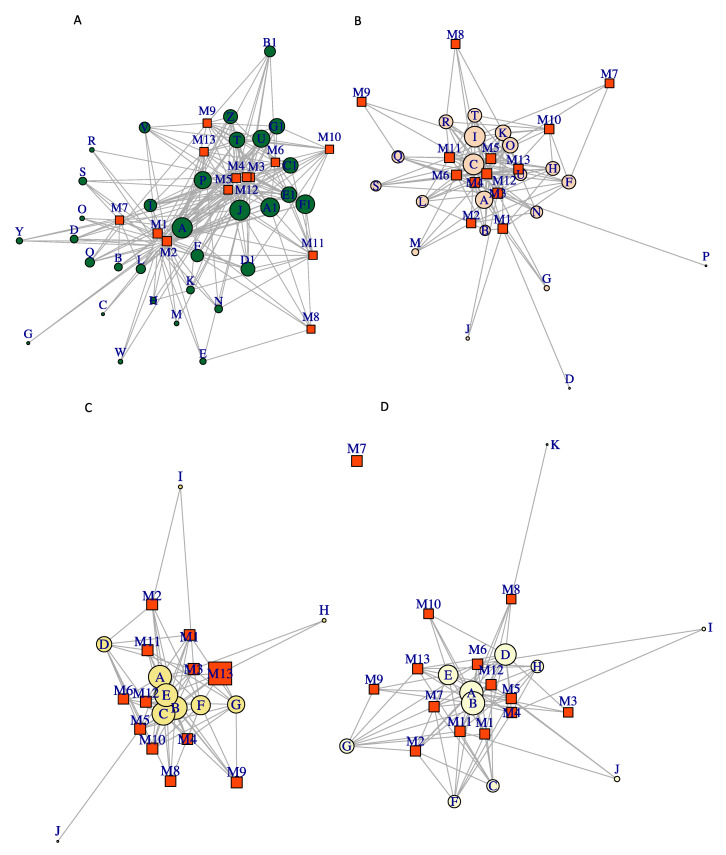
Co-occurrence network analysis by genus. The boxes represent the analyzed metagenomes and circle the species. (M1) Ching_S2_Mex, (M2) Ching_S1_Mex, (M3) ZNL_SRR10063240_34.5, (M4) ZNL_SRR10063241_30, (M5) ZNL_SRR10063242_27.2, (M6) CAN_SRR10095342_45, (M7) IN_SRR3050168_50, (M8) IN_SRR3961733_50, (M9) IN_SRR3961734_55, (M10) IN_SRR3961739_43, (M11) RSA_SRR5214706_50, (M12) JP_SRR7905022_60 (M13) IN_SRR816113699_52. (**A**) ***Rhodococcus* genus**. (A) *R. erythropolis*, (B) *R. erythropolis* SK121, (C) *R. erythropolis* DN1, (D) *R. erythropolis* PR4, (E) *R. erythropolis* CCM2595, (F) *R. qingshengii*, (G) *R. qingshengii* BKS 20-40, (H) *Rhodococcus* sp. 008, (I) *R. enclensis*, (J) *R. fascians*, (K) *Rhodococcus* sp. P27, (L) *Rhodococcus* sp. 311R, (M) *Rhodococcus* sp. ARP2, (N) *Rhodococcus* sp. ADH, (O) *Rhodococcus* sp. 164Chir2E, (P) *R. hoagii*, (Q) *Rhodococcus* sp. YH3-3, (R) *Rhodococcus* sp. BH4, (S) *Rhodococcus* sp. 66b, (T) *R. rhodochrous*, (U) *Rhodococcus* sp. RD6.2, (V) *Rhodococcus* sp. YL-1, (W) *Rhodococcus* sp. EPR-134, (Y) *Rhodococcus* sp. 1139, (Z) *Rhodococcus ruber*, (A1) *R. opacus*, (B1) *R. opacus* PD630, (C1) *Rhodococcus rhodnii*, (D1) *Rhodococcus* sp. AD45, (E1) *R. jostii*, (F1) *R. tukisamuensis*, (G1) *R. yunnanensis.* (**B**) ***Thiobacillus* genus**. (A) *Thiobacillus* sp. 65-29, (B) *T. denitrificans*, (C) *Thiobacillus* sp. 65-1059, (D) *Thiobacillus* sp. 63-78, (E) *Thiobacillus* sp. GWE1_62_9, (F) *T. thioparus*, (G) *Thiobacillus* sp. SCN 62-729, (H) *Thiobacillus* sp. SCN 63-374, (I) *Thiobacillus* sp. SCN 64-35, (J) *T. denitrificans* ATCC 25259, (K) *Thiobacillus* sp. 0-1251, (L) *Thiobacillus* sp. 65-1402, (M) *Thiobacillus* sp. SCN 64-317, (N) *Thiobacillus* sp. SCN 63-57, (O) *Thiobacillus* sp. SCN 63-1177, (P) uncultured Thiobacillus sp., (Q) *Thiobacillus* sp. 65-69. (**C**) ***Thiomonas* genus**. (A) *Thiomonas bhubaneswarensis*, (B) *Thiomonas intermedia*, (C) *Thiomonas* sp. FB-Cd, (D) *Thiomonas* sp. SCN 64-16, (E) *Thiomonas* sp. FB-6, (F) *Thiomonas* sp. CB2, (G) *Thiomonas* sp. CB3, (H) *Thiomonas* sp. CB6, (I) *Thiomonas* sp. ACO7, (J) *Thiomonas* sp. B1. (**D**) ***Bacillus* genus**. (A) *B. subtilis*, (B) *B. licheniformis*, (C) *B. cereus (D) B. cereus* R309803, (E) *B. mycoides*, (F) *B. wiedmannii*, (G) *Bacillus* sp. OxB-1, (H) *B. megaterium*, (I) *Bacillus* sp. F56, (J) *B. testis*, (K) *B. amyloliquefaciens group*, (M) *B. amyloliquefaciens*, (N) *B. salsus*, (O) *B. thuringiensis*, (P) *B. thuringiensis serovar israelensis* ATCC 35646, (Q) *B. weihenstephanensis*, (R) *B. anthracis*, (S) *B. gaemokensis*, (T) *B. manliponensis*, (U) *B. cytotoxicus.*

The *Rhodococcus* network showed a connectivity value of 0.54, weighted nestedness of 0.73, and modularity index of 0.39. The higher weighted closeness in the network was for *Rhodococcus erythropolis* (node A), with a value of 0.32. This result suggests that *R. erythropolis* is present in all circumneutral host springs; however, the value of weighted nestedness was closer for the Ching_S1_Mex and Ching_S2_Mex metagenomes, indicating the main predominance of this bacterium in metagenomes of Chignahuapan in comparison with other metagenomes. These results also confirm that *R. erythropolis* is the most abundant species (Figure 4A). 

The *Thiomonas* cluster showed the following network metric values: connectance 0.66; modularity index 0.10; weighted nestedness 0.65 (node B); *Thiomonas intermedia* and *Thiomonas* sp. FB-6 had the highest value-weighted closeness with 0.13 and 0.19, respectively. It was interesting that it was closer to most of the nodes in the graph or present in all terrestrial hot springs. In contrast, *Thiomonas* sp. CB6 (node H), *Thiomonas* sp. ACO7 (node I), and *Thiomonas* sp. B1 (node J) had the lowest value for weighted closeness, with 0.0067. Overall, in the Mexico metagenomes, *Thiomonas* species were less shared with the other metagenomes, indicating the microbial composition is particular (Figure 4D).

The *Acidithiobacillus* cluster showed a connectivity of 0.58, modularity index of 0.06, and weighted nestedness of 0.71. The weighted closeness lowest values were obtained for *Thiobacillus* sp. 0-1251 (node K) at 0.0014, *Thiobacillus* sp. SCN 64-35 (node I) at 0.002, and *T. denitrificans* ATCC 25259 (node J) at 0.007. Higher values of weighted closeness were determined for *Thiobacillus* sp. 65-29 (node A) at 0.43 and *T. denitrificans* (node B) at 0.20. These results indicate that all hot springs share *Acidithiobacillus* and highlight a particular species from this environment. In general, the most abundant species were *Thiobacillus* sp. 65-29 (node A), *T. denitrificans* (node B), *Thiobacillus* sp. 63-78 (node D), *Thiobacillus* sp. GWE1_62_9 (node E), mainly involved in the sulfur oxidation systems (Figure 4C).

The *Bacillus* cluster showed the following network metric values: connectance, 0.51; modularity index, 0.471; weighted nestedness, 0.47. Furthermore, the value-weighted closeness of most higher values was 0.13 for to *Bacillus cytotoxicus* (node U), *Bacillus subtilis* (node A) with 0.09, and *Bacillus cereus* (node C) with 0.09; these showed higher centrality or connections within the network in all metagenomes. The Chignahuapan samples were closer to *Bacillus licheniformis* (node B) at 0.06, whereas the lower weighted closeness was *B. cereus* R309803 (node D) at 0.0009, *Bacillus* sp. OxB-1 (node G) at 0.003, *B. amyloliquefaciens* (node M) at 0.006, *B. testis* (node J) at 0.001, and *Bacillus thuringiensis* serovar *israelensis* ATCC 35646 (node P) at 0.0009, which indicates that these species of bacteria are poorly connected in the network and are unique within the metagenomes Chig_S2_Mex (M1) and ZNL_SRR10063240 (M3). Interestingly, *Bacillus cereus* R309803 is a unique species in the Chignahuapan metagenome (Figure 4B). The value of modularity suggests that the network of *Bacillus* has a modular structure in this cluster. Modularity with values above 0.44 indicates that the networks are more connected. 

### 3.5. Functional Metagenomics Analysis 

**Figure 5:**
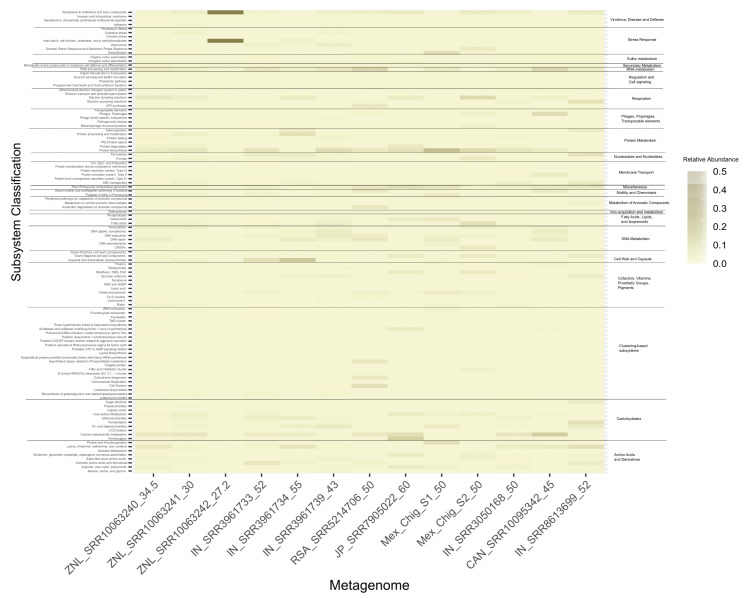
Functional annotation of circumneutral hot springs using the SEED subsystem database. On the x-axis are the different circumneutral metagenomes; on the y-axis is the classification into subsystems.

A functional analysis was performed with all metagenomes to determine whether the functional activity was similar in all the metagenomes; the amino acid sequences were annotated using SEED subsystems. The SEED subsystem is a classification system that organizes the coding sequences for functional categories into a hierarchy with 5 levels of resolution; in level 1, the families of proteins that share function. The results shown in Figure 5 correspond to the level 2 families.

The metabolism of carbohydrates (~13–22%) (central carbohydrate metabolism, CO_2_ fixation, and fermentation) are the hot springs’ main functional processes. From these, the metabolic pathways identified in high abundance correspond to the Calvin–Benson cycle, CO_2_ uptake carboxysome, Tricarboxylic acid (TCA) cycle, and oxidative phosphorylation pathways involved in CO_2_ fixation. Carbon fixation is a process where inorganic carbon (in the form of CO_2_) is transformed into organic compounds and is an essential process for the production of anabolism precursors. Four metabolic pathways in bacteria have the capacity of Carbon fixation: Calvin–Benson cycle, the reverse TCA cycle, the Wood–Ljungdahl pathway, and the 3-hydroxypropionate (3-HP) bicycle [75,76]. Two carbon fixation pathways (Calvin–Benson cycle and TCA cycle) were identified in the metagenomes, whereas only the gene encoding the ribulose bisphosphate carboxylase (RuBisCO) was found in the samples. The results suggest that the Calvin–Benson cycle is the primary metabolic pathway in the Chignahuapan terrestrial hot springs water ecosystem (Figure S4).

Carbonate was found in high concentrations in the hot water spring of Chignahuapan; the high abundance of HCO_3_ could correlate with the presence of pathways involved in the fixation of CO_2_, and the carbonic anhydrase, involved in the interconversion of CO_2_ to carbonate [77]. Additionally, the CO_2_ is dissolved in water and can form different compounds such as carbon dioxide, carbonic acid, bicarbonate, and carbonate, and sulfur-oxidizing bacteria can fix it. In this context, *Acidithiobacillus ferrooxidans* was found in metagenomes and uses CO_2_ for growth [78]. Furthermore, other thermophilic bacteria have been reported, including *Bacillus schlegelii* and *P. thermocarboxydovorans* growing with CO_2_ as a unique carbon and energy source [79,80]. 

At the first level within the SEED category, the amino acids and derivatives, cofactors, vitamins, prosthetic groups, and pigments were the most abundant categories (~2–14%), followed by lysine, threonine, methionine, and cysteine biosynthesis. Most of these amino acids involved in protein synthesis and as cofactors in many metabolic reactions; however, methionine and cysteine biosynthesis are involved in the sulfur metabolism for biosynthesis of sulfur-containing biomolecules, such as the cysteine sulfinate-dependent pathways to produce sulfite or turine [81]. 

This metabolic category has also been reported in abundance in the hot springs of the Araró region, located in the Trans-Mexican Volcanic Belt [32], suggesting a central role in the biosynthesis of diverse compounds, for assimilatory sulfate reduction, SOX pathway, and for obtaining energy in thermal water environments (Figure 5).

Sulfur metabolism has been reported in microbial mats, hydrothermal vents, and YNP hot springs, where microorganisms called sulfur oxidizers live, as well as many chemolithotrophic Proteobacteria [77,82]. Overall, in the metagenomes analyzed, the proportion of sulfur metabolism was present in the metagenomes (0.9–1.9%). 

Chignahuapan hot springs contain the complete SOX pathway. This pathway is involved in the oxidation of sulfide (S^2−^) and thiosulfate (S_2_O_3_^2−^) to sulfate (SO_4_^2−^) (Figure S5). This pathway has been reported present in alpha and epsilon-proteobacteria [18,59,83], identified in the two metagenomes of Puebla, suggesting that *epsilon-proteobacteria* are contributing to sulfide and thiosulfate oxidation, as an energy source. It has been reported that some bacteria can remove inorganic sulfur from oil, and it has been investigated that the enzymes to carry out this process are within the sox pathway; this was characterized in *Rhodococcus* sp. strain IGTS8 [84], the most abundant genera identified in the hot springs. The bacteria *H. neapolitanus* and *Acidithiobacillus caldus* have sulfur-oxidizing enzyme systems involved in the SOX pathway, and both bacteria were present in Chignahuapan.

In summary, sulfur oxidation is carried out by bacteria within the phylum Proteobacteria and Actinobacteria in Chignahuapan thermal springs. Where the assimilatory sulfate pathway involved in the reduction in sulfate (SO_4_^2−^) to sulfide (S^2−^) was complete (Figure S5) [85,86]. These results contrast with bacteria involved in sulfur metabolism such as Deltaproteobacteria and Firmicutes found in Yellowstone National Park [87,88]. 

Nitrogen fixation is associated with carbon fixation in the microbial mat communities, and the nitrogen fixation occurred in many environments [89]. Nitrogen-fixing enzymes could be expected to be present in metagenomes. However, in general, the nitrogen fixation pathway enzymes found to be abundant in the samples of Chignahuapan were the enzymes involved with the pathway assimilatory nitrate reduction nitrate to ammonia (*nasA* and *nirB* genes) and ammonia-lyases also involved in ammonia production. As mentioned above, this pathway is associated with nitrogen fixation, and carbonic anhydrase was found here, which is associated with converting carbon dioxide to carbonates (Figure S6).

The stress oxidative and heat shock category was highly abundant in Chignahuapan of the reactive oxygen species (ROS) and can cause irreversible damage to cells, and indifferent thermophilic bacteria, and it has been reported to present a superoxide dismutase; this enzyme catalyzes the dismutation of the superoxide (O_2_^1−^) into oxygen (O_2_). In the case of the samples, the bacteria that presented the putative enzyme were *Thioalkalivibrio* spp., and *Acinetobacter* spp., while *Rhodococcus opacus* B4 had NrdH, which mediates resistance to oxidative stresses. Previously, bacteria with superoxide dismutases had been reported, such as *Aquifex pyrophilus*, *Hydrogenobacter thermophilus*, *Thermus thermophilus*, *Propionibacterium shermanii*, and *Rhodothermus* sp. among others. This system is essential to avoid damage to extreme environments [90]. This abundant category of functions also relates to other thermal environments, such as the mats from Araró Mexico [32]. 

Similarly, as there are mechanisms to prevent cell damage, there are DNA repair mechanisms; previously in hypersaline waters, we observed that microorganisms have different enzymes involved in DNA repair [58]. In the case of thermophilic microorganisms, there are efficient mechanisms to prevent DNA repair and proteins and the lipid membrane from preventing these damages: for example, the composition of fatty acids changes with increasing temperature [90]. The thermophilic microorganisms of Chignahuapan also have repair systems; however, they are abundantly presented with the enzyme exonuclease SbcC, which is involved in DNA repair when alkylation is damaged.

Overall, the annotation allowed us to predict the functional potential of the thermophilic community of circumneutral metagenomes. Whereas mainly fixation of carbon is the crucial pathway and amino acids and derivatives, many of them contribute to sulfur metabolism and fixation of carbon. Likewise, the pathway to oxidation and reduction in metabolites of sulfur was completed. Many of the thermophilic microorganisms have a mechanism to prevent oxidative stress damage and repair DNA damage.

### 3.6. Viral Community Composition

For the viral community in a circumneutral terrestrial hot spring, the analysis shows that overall, nine viral families were retrieved, and many of them infect bacteria, such as Siphoviridae, Myoviridae, Podoviridae, Corticoviridae, and Herelleviridae. These viruses also infect invertebrates Baculoviridae, eukaryotic algae Phycodnaviridae, and Protozoan Mimiviridae. 

**Figure 6:**
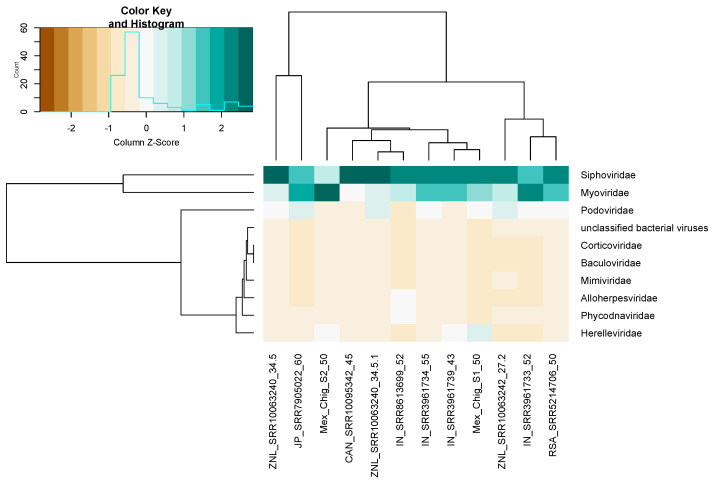
Heat map abundance of viral families retrieved from Vibrant. Myoviridae and Siphoviridae families were ubiquitous in metagenomes.

Clustering analysis revealed that most abundant families are Myoviridae and Siphoviridae, which are ubiquitous in all metagenomes. These samples also showed that the metagenomes of Mexico can be grouped with samples from India and Canada with similar temperatures (Figure 6). 

These results contrast with previous reports in thermal or geothermal terrestrial environments; it has been determined that the main families of viruses present are Fuselloviridae, Bicaudaviridae, Turriviridae, Ampullaviridae, Guttaviridae, Lipothrixviridae, Rudiviridae, and Globuloviridae [3,91]. Most of these virus families infect Archaea that live in thermal environments with a temperature above 80 °C, and these viruses have been called Archeoviruses [92,93]. 

However, it was expected that the viruses recovered in these metagenomes mainly infect bacteria, since the temperature and pH are also moderate in the analyzed metagenomes. Thus, the dominance of viruses that infect prokaryotes found are moderate thermophilic bacteria. Nevertheless, in the hot springs of Chignahuapan, besides Siphoviridae and Myoviridae, the Herelleviriadae family was abundant.

The Herelleviriadae family was recently described: the phylogenetic evidence performed at the genome and proteome level, and by a single gene (capsid, tail protein, DnaB395 like-helicase), showed that these viruses are polyphyletic since they are grouped into five different clades or subgroups. This family contains linear viral genomes, with a length of 125–170 kbp, that infect the genus *Bacillus* [94]. 

The abundance of bacteriophages of the Herelleviriadae family correlates with the taxonomic assignment results, where the genus *Bacillus* was present in both metagenomes, and according to the co-occurrence analysis, the main associated species of the metagenomes of Mexico were mainly *B. subtilis* and *B. licheniformis*. 

VIRSorter and VIBRANT programs were used in order to retrieve viral contigs. This software failed to recover the complete genome from samples of Chignahuapan, only partial sequences, probably because of the low performance in the metagenome sequencing. However, according to the taxonomic classification from viral contig retrieval, the most abundant viruses were *Acidithiobacillus* phage AcaML1, *Bacillus* phage SIOphi, *Bacillus* virus Bobb, *Acinetobacter* virus R3177, and *Bacillus* phage Shbh1 in the samples from Mexico, correlating with what was found in bacteria. According to the taxonomic assignment, 200 viruses were recovered with Vibrant and 125 viruses with Virsorter in all samples. In the Japanese metagenome, there was a greater abundance of viruses (Figure S7).

Overall, the viral communities in moderate thermophilic environments that infected moderate to thermophilic bacteria demonstrated distinct viral community structures among the circumneutral thermal springs, compared with acid or hyperthermophile hot springs such as Yellowstone [92]. These results indicate that the circumneutral thermal springs harbor viral communities with phage double-stranded DNA that infect mainly bacteria, followed by viruses that infect invertebrates or eukaryotic algae.

Through an analysis of occurrence carried out with the recovered viruses from Vibrant, the results from the analysis revealed there are few connections between virus species in the metagenomes, each having many particular species. These results correlate with the classifications of bacteria, where each one observed that each environment has a genus of particular bacteria, and that terrestrial hot springs have a common *Acidithiobacillus phage* AcaML1, which infects *Acidithiobacillus caldus* (Figure S8). 

*Acidithiobacillus caldus* has been reported in the various thermal environments. It is a moderately thermoacidophilic bacteria that contributes to the carbon and sulfur cycles, as it obtains energy from the oxidation of elemental sulfur for carbon dioxide fixation, and it is ubiquitous in sulfide mineral environments [95].

### 3.7. Auxiliary Metabolic Genes and Whole Viral Genomes 

We evaluated the presence of AMGs in the viral contigs recovered from all the metagenomes, but in the case of the Chignahuapan metagenomes, no AMGs were obtained. The viral genes recovered in these correspond mainly to structural parts of the virion. Thus, this comparison was carried out only with those viral contigs that contained AGMs.

The AMGs have been related to an increase in the fitness and altering or complementing of their host’s metabolism, facilitating adaptation under adverse conditions [96,97,98], and they have been related to photosynthesis, carbon fixation [99], and sulfur and nitrogen biogeochemical cycles [100]. 

**Figure 7:**
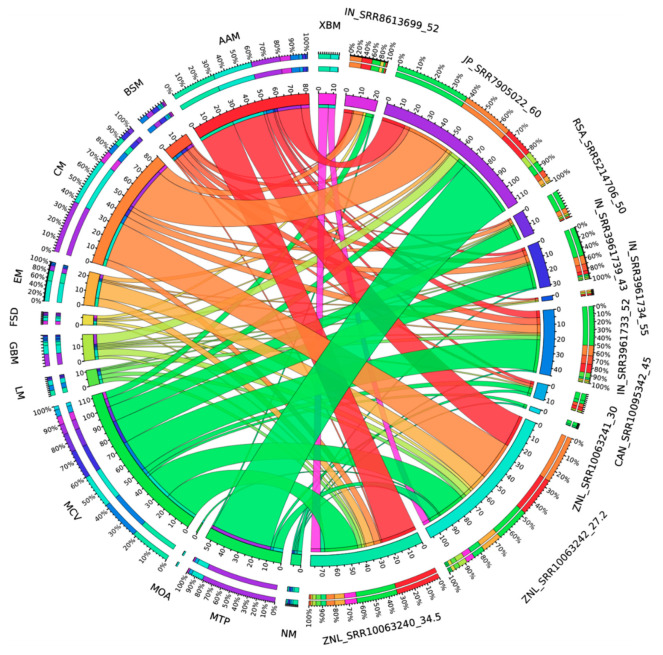
Auxiliary metabolic genes recovered from Vibrant. Representation of the metagenomes that contain the viral AMG. AAM: Amino acid metabolism; GBM: Glycan biosynthesis and metabolism; MCV: Metabolism of cofactors and vitamins; MTP: Metabolism of terpenoids and polyketides; BSM: Biosynthesis of other secondary metabolites; NM: Nucleotide metabolism; CM: Carbohydrate metabolism; LP: Lipid metabolism; EM: Energy metabolism; FSD: Folding, sorting and degradation; XBM: Xenobiotics biodegradation and metabolism; MOA: Metabolism of other amino acids.

The most abundant AMGs were classified within the functional categories metabolism of cofactors and vitamins (MCV), carbohydrates metabolism (CM), amino acids metabolism (AAM), metabolism of terpenoids, and polyketides (Figure 7). 

In the MCV category, the virus contributes to the folate biosynthesis pathway (KEGG entries identified: K00287, K01495, K01737, K06920, K09457, K10026). Tetrahydrofolate is a cofactor present in all bacteria, and it is essential to the growth; synthesis of formylmethionyl tRNAfMet, carried out by the enzyme dihydrofolate reductase (DHFR); and in thermophilic bacteria, has been identified to use modified folates [101], and within the folate biosynthesis pathway, the AMG 7-*cyano*-7-*deazaguanine* synthase was also found, a hypermodified 7-deazaguanosine, which has been previously reported in viruses; it is proposed that phages have taken the 7-deazaguanine from bacteria to evade the restriction-modification system (RM system) [101,102]. Interestingly, viruses found in thermophilic environments have these AGMs. 

Additionally, other metabolic pathways were found within this category MCV such as Thiamine metabolism (K03153, K04487), biotin metabolism (K00059, K09458), pantothenate and coenzyme (CoA) biosynthesis (K13038), and retinol metabolism (K11153), and within nicotinate and nicotinamide metabolism (K00858, K01916, K03462, K13522) viruses have a gene that could modify or complement these metabolic pathways. 

In the category carbohydrate metabolism (CM), AMGs were involved in the pentose phosphate pathway (K00616, K01053), galactose metabolism (K01784), ascorbate and alternate metabolism (K00012), propanoate metabolism (K00822), and glyoxylate and dicarboxylate metabolism. For example, in the case of the phosphate pathway transaldolase (*talC* gene), it catalyzes glyceraldehyde-3-phosphate, sedoheptulose-7-phosphate into fructose-6-phosphate and erythrose-4-phosphate. This class of transaldolases has been reported in cyanomyoviruses [97]. 

The categories least abundant but not less interesting, are those for obtaining energy and degradation of hydrocarbons. Some viruses have been reported in oil reserves, which could have interesting functions with the biotechnological applications [103].

The energy category was found in a low proportion; the pathways involved were sulfur metabolism and methane metabolism. One of the most important enzymes that we found was *cysH* phosphoadenosine phosphosulfate reductase, which is involved in the reduction in 3′-phosphoadenosine-5′-phosphosulfate (PAPS) to sulfite, an intermediate step of the pathway assimilatory sulfate reduction. Additionally, *sufS* which encodes cysteine desulfurase was found: this enzyme mobilizes the sulfur from L-cysteine. 

In previous work, it has been reported that the viruses inside hydrothermal vents carry out AMG related to dissimilatory sulfite; specifically, the enzyme reverse dissimilatory sulfite reductases encoded by *rdsrA* and *rdsrC* in charge of oxidizing elemental sulfur, and some oxidizing sulfur bacteria lack the Sox system and use *rdsrA* for oxidizing elemental sulfur [104]. Nevertheless, here, the AMGs found were *cysH* and *sufS* involved in sulfur metabolism. Therefore, viruses and bacteria present in circumneutral environments contribute to the sulfur cycle.

Finally, there are some bacteria that are capable of carrying out the degradation of hydrocarbons; interestingly, we found viral AMGs in low abundance involved in the degradation of xenobiotics: for example, in the Benzoate degradation pathway (K01055), *pcaD* genes encoding to 3-oxoadipate enol-lactonase. Toluene degradation and fluorobenzoate degradation pathway (K01061) were found through the hydrolase carboxymethylenebutenolidase. This represents a biotechnologically important finding since these sequences could be obtained for use in the industry. These results reveal that viruses contribute to carbohydrate metabolism, sulfur cycles, and the degradation of aromatic compounds.

## 4. Conclusions

The composition of microorganisms in Chignahuapan is driven by chemical composition and geographic location. Since the microbial community’s structure was particular where the bacteria of the genera *Rhodococcus*, *Acinetobacter*, *Thermomonas*, *Tepidimonas*, and *Azotobacter* predominated, in comparison to other circumneutral environments, these bacteria are sulfur oxidizers, which is consistent with the functional analysis where the sulfur reduction and oxidation pathways were complete. The functional analysis also predicted that the Calvin–Benson cycle metabolic pathways are the main pathways to contribute to carbon fixation. Furthermore, the microorganisms present in circumneutral environments have mechanisms that prevent cellular and DNA damage. Therefore, the microbial community structure in particular in each location is driven by physicochemical properties, but many metabolic pathways were common in circumneutral terrestrial hot springs, which contribute to carbon fixation. In the hot springs’ viral community, prokaryotic viruses predominate overall, but in Chignahuapan, the Herelleviridae family was mainly abundant. The analysis of the auxiliary genes revealed that the viruses also contribute to metabolic pathways and the sulfur cycle. This study shows the information on the microbial and viral diversity in Mexican hot springs and compares these microbial communities with other metagenomic samples, thus providing an opportunity to understand the role of the viral AMGs and the structure of the viruses in the adaptation process to circumneutral hot springs. Additionally, this report serves as a reference to viromes in extremophile environments from Mexico.

## Figures and Tables

**Table 1:** Physicochemical parameters of the Chignahuapan hot springs.

Chemical Properties	Mex_Chig_S1	Mex_Chig_S2
Temperature in °C	49–50	45
pH	7.02	6.66
Electrical conductivity dS m^−1^	1.52	1.50
Ca^2+^ mg L^−1^	203.1	81.6
Mg^2+^ mg L^−1^	33.2	17.3
Na^1+^ mg L^−1^	102.0	212.9
K^1+^ mg L^−1^	14.6	12.8
NO_3_^−1^ mg L^−1^	16.9	12.2
SO_4_^−2^ mg L^−1^	25.6	30.2
PO_4_^−3^ mg L^−1^	4.2	2.5
CO_3_^−2^ mg L^−1^	0	0
HCO_3_^−1^ mg L^−1^	780.8	634.4
Cl^−1^ mg L^−1^	196.0	98.9

**Table 2:** Total of contigs classified by Kaiju of Metagenome from Chignahuapan.

Sample	No. Total of Contigs	Total Contigs		% Contigs Classified within Domain			
		Classified	Unclassified	Bacteria	Archaea	Eukarya	Virus
Mex_Chig_S1	8474	8082 (95.5%)	392 (4.51%)	91.8%	1.8%	0.802%	1.08%
Mex_Chig_S2	8361	7645 (91.4%)	716 (8.56%)	88.4%	1.3%	0.7299%	0.957%

## Supplementary Materials

The following are available online at https://www.mdpi.com/2076-2607/8/11/1677/s1, Figure S1: Taxonomic profiling and community structure comparison at the domain level. Our profiling showed a dominance of Bacteria, at around 88.4 to 91.8%, followed by Archaea, viruses, and eukaryotes; Figure S2: Stack plots of the relative abundances of Archaea and Bacterial at the level of phylum. (A) Archaea domain. The Euryarchaeota phylum was dominant in the samples. (B) Bacteria domain. The Proteobacteria phylum was dominant in the samples; Figure S3: Biplot showing an association of genera based on location. The samples are grouped based on location; Figure S4: Pathway carbon metabolism from Chignahuapan. Colors indicate enzymes present in metagenomes; Figure S5: Pathway sulfur metabolism from Chignahuapan the green color indicates the enzymes present in the metagenome; Figure S6: Pathway nitrogen metabolism from Chignahuapan. Colors indicate enzymes present in metagenomes; Figure S7: Species of virus identification (A) Vibrant and (B) Virsorter; Figure S8: Virus network analysis. Metagenomes (M1) ZNL_SRR10063242_27.2, (M2) JP_SRR7905022_60, (M3) IN_SRR3961734_55, (M4) Mex_Chig_S1, (M5) ZNL_SRR10063241_30, (M6) ZNL_SRR10063240_34.5, (M7) IN_SRR3961739_43, (M8) RSA_SRR5214706_50, (M9) IN_SRR3961733_52, (M10), IN_SRR8613699_52, (M11) CAN_SRR10095342_45, (M11) Mex_Chig_S2. Virus species (1) Bacillus virus Bobb, (2) Bacillus virus Bcp1, (3) Nitunavirus, (4) Siminovitchvirus, (5) Enterobacteria phage vB_KleM-RaK2, (6) Bixzunavirus, (7) Acidovorax virus ACP17, (8)Sinorhizobium virus M12, (9) Pectobacterium phage CBB, (10) Campylobacter virus Los1, (11) Campylobacter phage PC5, (12) Enterobacter phage Arya, (13) Pseudomonas phage PPpW-3, (14) Erwinia phage vB_EamM_Parshik, (15) Acinetobacter virus ME3, (16) Acinetobacter virus LZ35, (17) Burkholderia phage KS5, (18) Burkholderia phage vB_BceM_AP3, (19) Ralstonia phage RSY1, (20) Stenotrophomonas phage Smp131, (21) Pseudoalteromonas phage C5a, (22) Agrobacterium virus Atuph07, (23) Svunavirus, (24) Enterobacter virus PG7, (25) unclassified Tequatrovirus, (26) Yersinia virus PST, (27) Acinetobacter phage Acj9, (28) Morganella phage vB_MmoM_MP1, (29) Acidithiobacillus phage AcaML1, (30) Acinetobacter phage Ab105-1phi, (31) Alteromonadaceae phage B23, (32) Aurantimonas phage AmM-1, (33) Bacillus phage AR9, (34) Bordetella phage vB_BbrM_PHB04, (35) Bradyrhizobium phage BDU-MI-1, (36) Caulobacter phage Cr30, (37) Cyanophage S-RIM12, (38) Cyanophage S-RIM14, (39) Cyanophage S-RIM32, (40) Cyanophage S-RIM44, (41) Deep-sea thermophilic phage D6E, (42) Faecalibacterium phage FP_Mushu, (43) Lake Baikal phage Baikal-20-5m-C28, (44) Ochrobactrum phage POA1180, (45) Prochlorococcus phage P-HM1, (46) Prochlorococcus phage P-SSM2, (47) Prochlorococcus phage P-SSM7, (48) Prochlorococcus phage P-TIM68, (49) Pseudomonas phage Lu11, (50) Pseudomonas phage PaBG, (51) Rhizobium phage vB_RleM_PPF1, (52) Salicola phage SCTP-2, (53) Shewanella phage SFCi1, (54) Shewanella sp. phage 1/40, (55) Shigella phage SfIV, (56) Stenotrophomonas phage IME-SM1, (57) Stenotrophomonas phage vB_SmaS-DLP_6, (58) Synechococcus phage ACG-2014f, (59) Synechococcus phage ACG-2014g, (60) Thermus phage TMA, (61) Xanthomonas phage XacN1, (62) Yersinia phage phiR1-37, (63) Edwardsiella virus MSW3, (64) Enterobacteria phage J8-65, (65) Pseudomonas phage vB_PaeP_PAO1_Ab05, (66) Ralstonia phage RS-PII-1, (67) Ralstonia phage RsoP1EGY, (68) Rhizobium phage RHEph01, (69) Erwinia phage vB_EamP-S2, (70) Escherichia virus Pollock, (71) Erwinia virus Frozen, (72) Pseudomonas virus KPP25, (73) Escherichia phage APC_JM3.2, (74) Bordetella virus BPP1, (75) Enterobacter phage Tyrion, (76) Aeromonas phage phiARM81mr, (77) Agrobacterium phage Atu_ph08, (78) Burkholderia phage vB_BmuP_KL4, (79) Cellulophaga phage phi46:3, (80) Cellulophaga phage phi14:2, (81) Delftia phage RG-2014, (82) Pseudoalteromonas phage HP1, (83) Pseudomonas phage AF, (84) Pseudomonas phage TC6, (85) Pseudomonas phage ZC08, (86) Puniceispirillum phage HMO-2011, (87) Ralstonia phage DU_RP_II, (88) Ralstonia phage RSK1, (89) Sinorhizobium phage PBC5, (90) Sinorhizobium phage phiM5, (91) Xanthomonas citri phage CP2, (92) Xylella phage Xfas53, (93) Burkholderia virus AH2, (94) Streptomyces phage Maneekul, (95) Mycobacterium virus Vincenzo, (96) Mycobacterium virus Godines, (97) Mycobacterium phage 40BC, (98) Pseudomonas phage JBD18, (99) Gordonia virus Bowser, (100) Gordonia virus Britbrat, (101) Pseudomonas phage MP42, (102) Vibrio virus pVp1, (103) Rhodobacter virus RcCronus, (104) Stenotrophomonas virus DLP5, (105) Pseudomonas phage phi1, (106) Doucettevirus, (107) Mycobacterium virus Pukovnik, (108) Mycobacterium virus Timshel, (109) Mycobacterium phage HINdeR, (110) Escherichia phage ST2, (111) Microbacterium virus Koji, (112) unclassified Laroyevirus, (113) Vibrio virus MAR10, (114) Marvinvirus, (115) Dinoroseobacter virus D5C, (116) Mycobacterium virus Panchino, (117) Pseudomonas virus PaMx25, (118) Pseudomonas phage JG012, (119) Gordonia virus Zirinka, (120) Gordonia phage BatStarr, (121) Pseudomonas phage AAT-1l, (122) Xanthomonas phage Xoo-sp2, (123) unclassified Pbi1virus, (124) Escherichia phage YDC107_1, (125) Streptomyces virus Jay2Jay, (126) Gordonia virus OneUp, (127) Burkholderia phage Bcep176, (128) Burkholderia phage KS9, (129) unclassified Timquatrovirus, (130) Arthrobacter phage vB_ArS-ArV2, (131) Azospirillum phage Cd, (132) Bacillus phage vB_BhaS-171, (133) Bifidobacterium phage Bbif-1, (134) Caulobacter phage CcrColossus, (135) Caulobacter phage Sansa, (136) Cellulophaga phage phi19:1, (137) Corynebacterium phage LGCM-V4, (138) Croceibacter phage P2559Y, (139) Erysipelothrix phage phi1605, (140) Geobacillus virus E2, (141) Geobacillus virus E3, (142) Gordonia phage Confidence, (143) Gordonia phage GMA1, (144) Gordonia phage GMA2, (145) Gordonia phage McGonagall, (146) Halomonas phage QHHSV-1, (147) Klebsiella phage 5 LV-2017, (148) Lactobacillus phage PLE3, (149) Lactococcus phage PLg-TB25, (150) Microbacterium phage Paschalis, (151) Pseudomonas phage JBD25, (153) Pseudomonas phage JBD44, (154) Pseudomonas phage JBD68, (155) Pseudomonas phage phiPSA1, (156) Pseudomonas phage PS-1, (157) Pseudomonas phage YMC11/02/R656, (158) Psychrobacter phage Psymv2, (159) Ralstonia phage RS138, (160) Rhizobium phage 16-3, (161) Rhizobium phage vB_RleS_L338C, (161) Rhodobacter phage RcapMu, (162) Rhodococcus phage Jace, (163) Rhodovulum phage vB_RhkS_P1, (164) Sinorhizobium phage phi2LM21, (165) Sinorhizobium phage phi3LM21, (166) Sinorhizobium phage phiLM21, (167) Stenotrophomonas phage S1, (168) Streptococcus phage phiZJ20091101-1, (169) Streptomyces phage Chymera, (170) Streptomyces phage Ibantik, (171) Streptomyces phage mu1/6, (172) Synechococcus phage S-CBS3, (173) Synechococcus virus S-ESS1, (174) Thiobacimonas phage vB_ThpS-P1, (175) Paenibacillus phage Dragolir, (176) Paracoccus virus Shpa, (177) Acinetobacter virus R3177, (178) Acinetobacter phage Ab105-2phi, (179) Acinetobacter phage vB_AbaS_TRS1, (180) Gordonia virus Vivi2, (181) Roseobacter virus RDJL1, (182) Pseudomonas virus LKO4, (183) Pseudomonas virus MP1412, (184) Pseudomonas virus PAE1, (185) Bordetella phage FP1, (186) Pseudomonas phage AN14, (187) Pseudomonas phage vB_PaeS_S218, (188) Marinomonas phage YY, (189) Cyprinid herpesvirus 2, (190) Ictalurid herpesvirus 1, (191) Megavirus chiliensis, (192) Moumouvirus, (193) Tupanvirus soda lake, (194) Paramecium bursaria Chlorella virus 1, (195) Ostreococcus tauri virus 2, (196) Yellowstone lake phycodnavirus 1, (197) Yellowstone lake phycodnavirus 2, (198) Campylobacter phage A18a, (199) Synechococcus phage S; Table S1: Metagenome data considered for the analysis.
