# Supplementary material for: The Microbial Composition in Circumneutral Thermal Springs from Chignahuapan, Puebla, Mexico Reveals the Presence of Particular Sulfur-Oxidizing Bacterial and Viral Communities
Source: Microorganisms. 2020 Oct 29;8(11):1677. doi: 10.3390/microorganisms8111677 (PMC7692377; doi:10.3390/microorganisms8111677)
Supplement: Supplementary file 1 [file microorganisms-08-01677-s001.pdf]

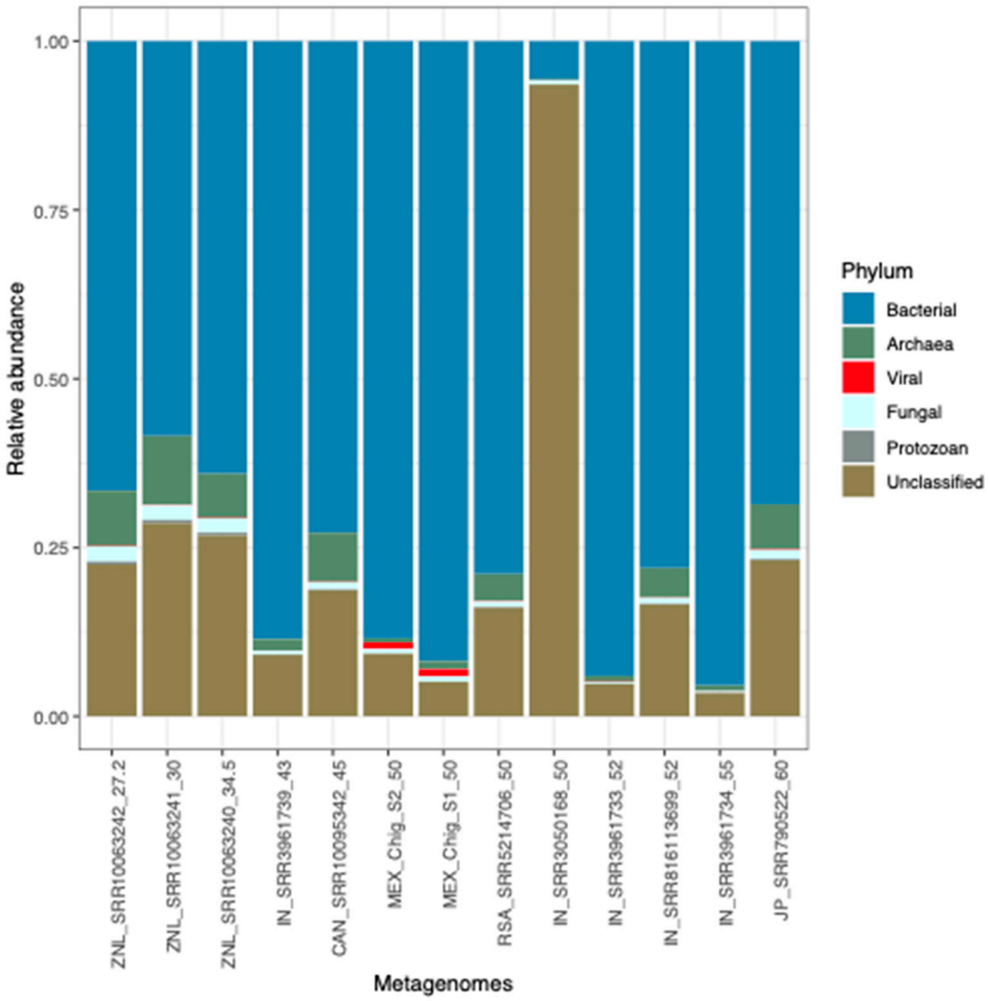

Figure S1. Taxonomic profiling and community structure comparison at the domain level. Showed a dominance of Bacteria around 88.4% to 91.8%, followed by Archaea, Viruses, and Eukaryotes. .

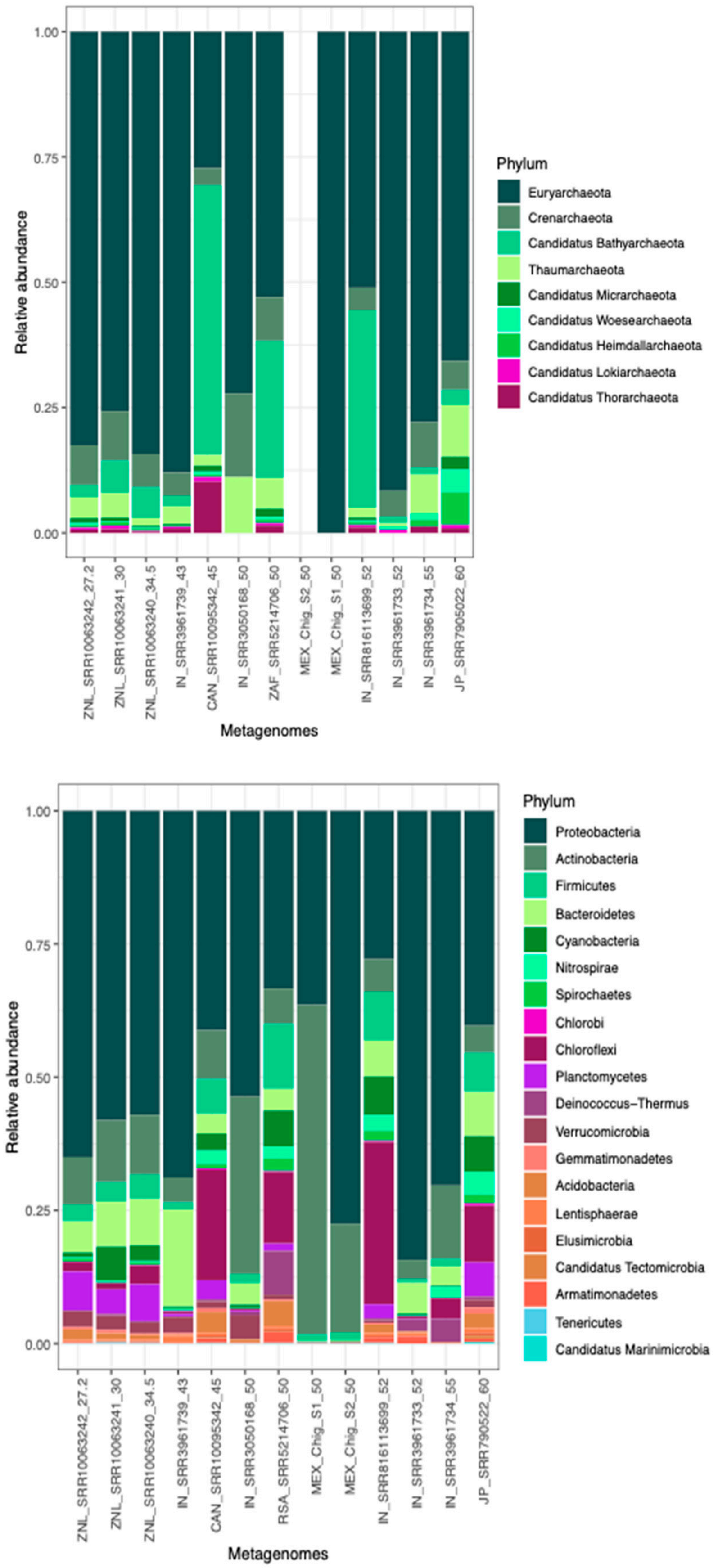

Figure S2. Stack plots the relative abundances of Archaea and Bacterial at the level of phylum. A) Archaea domain. Euryarchaeota phylum was dominant in the samples B) Bacteria domain. Proteobacteria phylum was dominant in the samples.

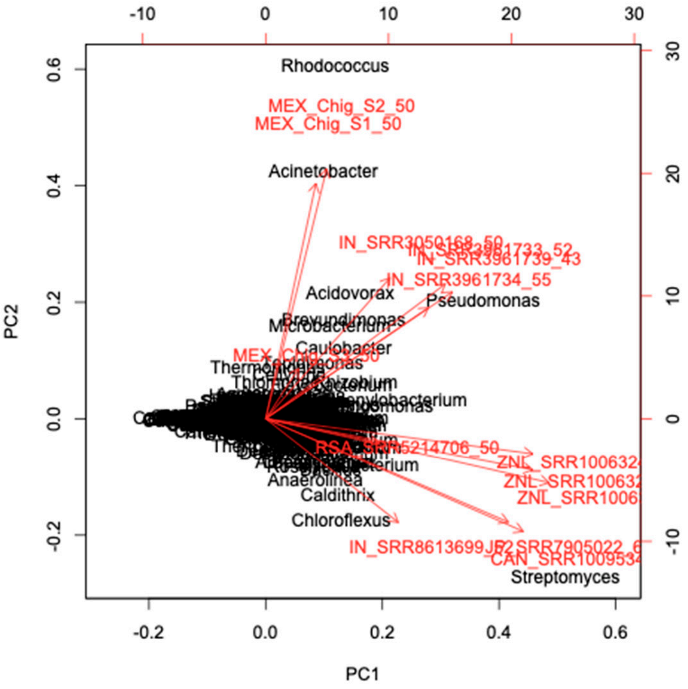

Figure S3. Biplot showing an association of genera based on location. The samples are grouped using location.

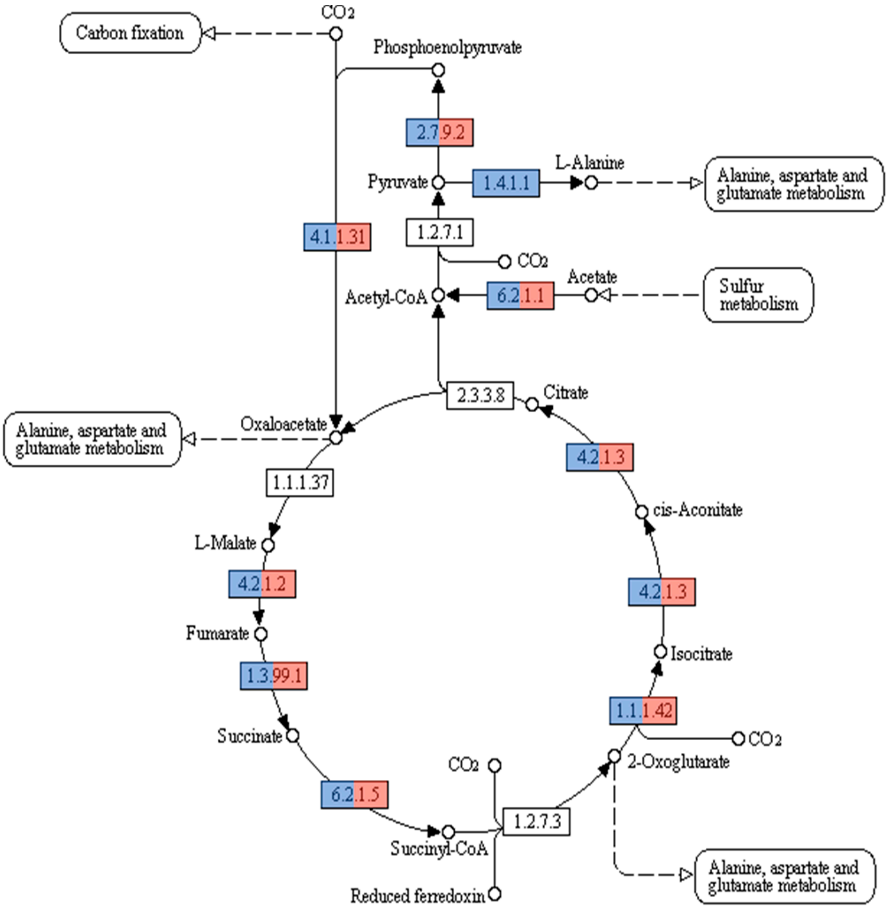

Figure S4. Pathway Carbon metabolism from Chignahuapan. Colors indicate enzymes present in metagenomes.

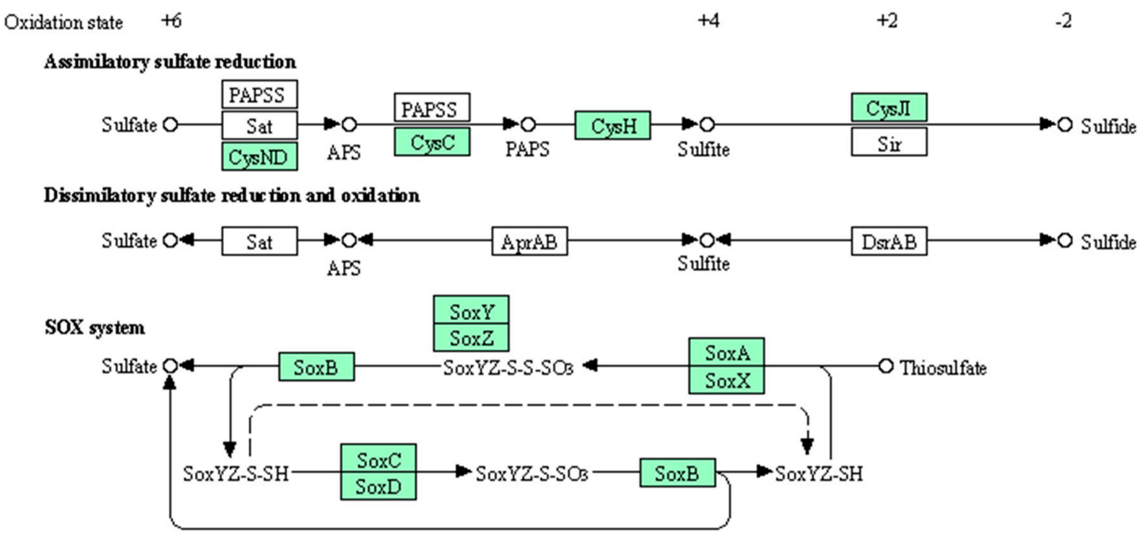

Figure S5. Pathway Sulfur metabolism from Chignahuapan. the green color indicates the enzymes present in the metagenome.

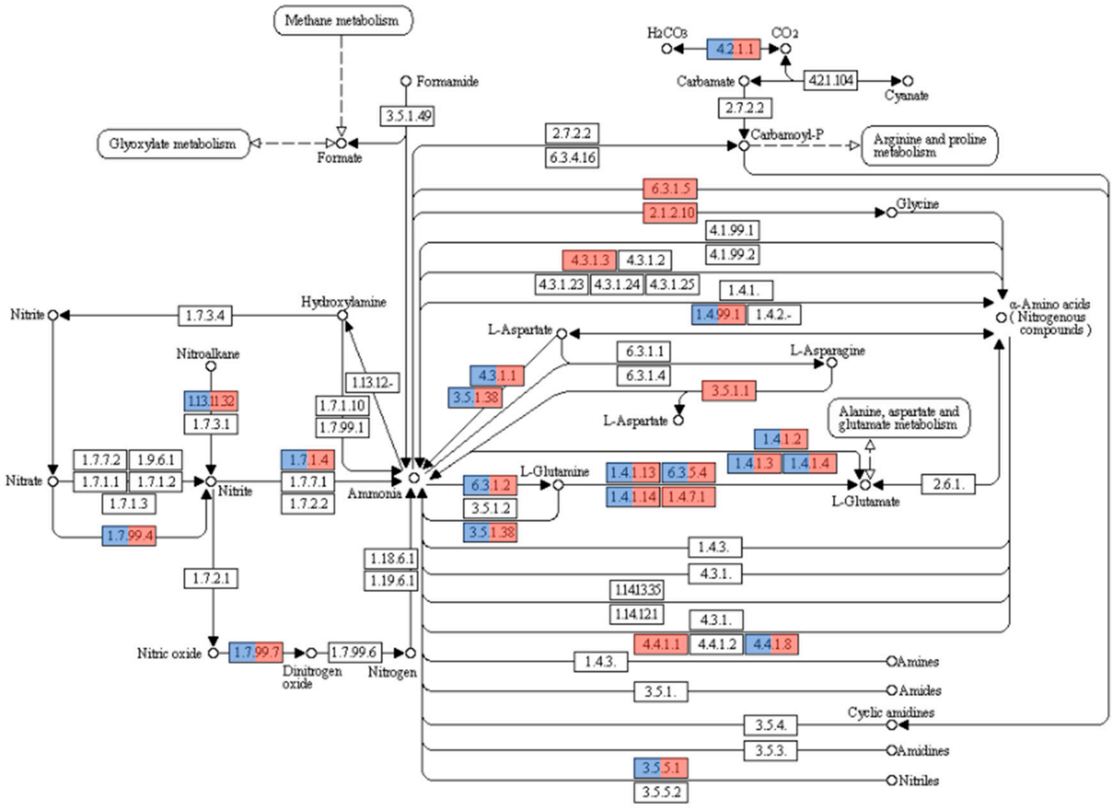

Figure S6. Pathway Nitrogen metabolism from Chignahuapan. Colors indicate enzymes present in metagenomes.

A

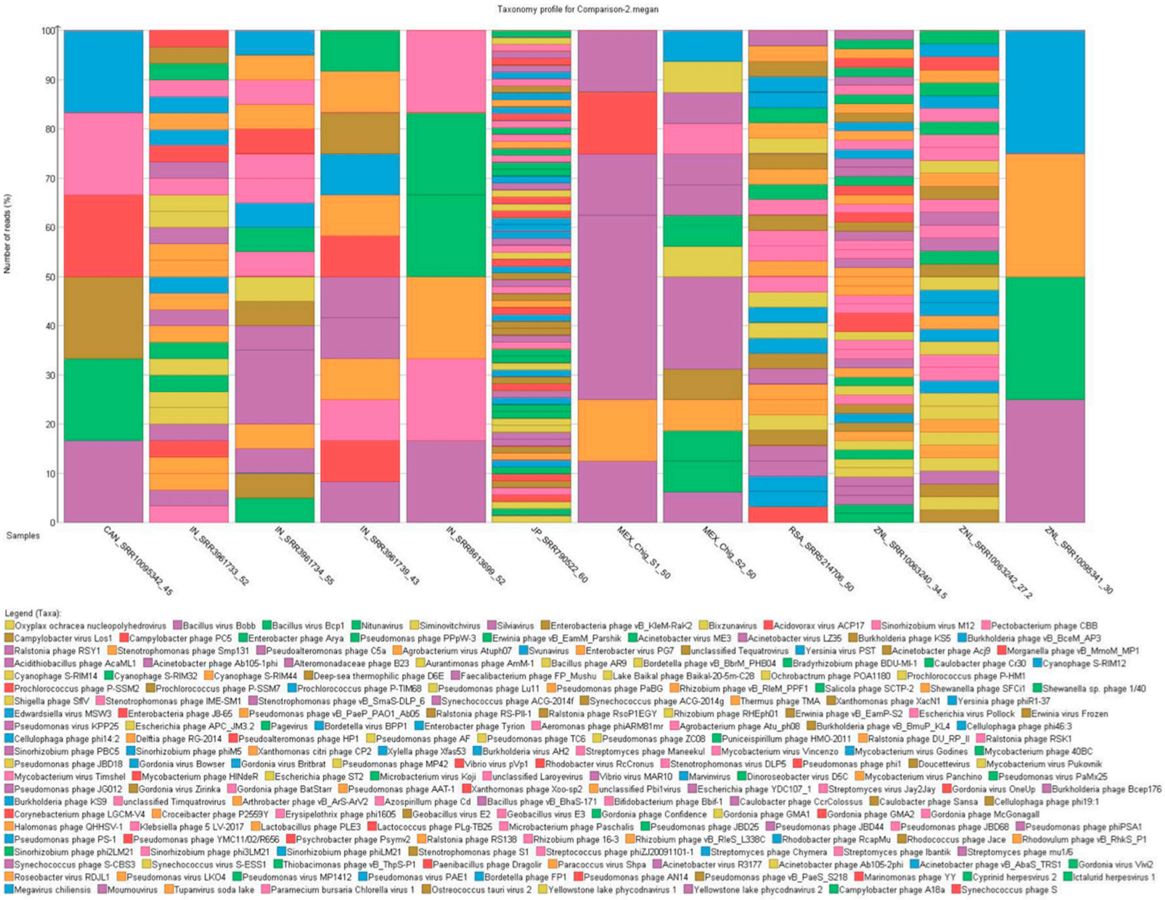
Taxonomy profile for Comparison-2.megan
Number of reads (%)
Samples
CAN_SRR10095342_45
IN_SRR3961733_52
IN_SRR3961734_55
IN_SRR3961739_43
IN_SRR8613699_52
JP_SRR790522_60
MEX_Chig_S1_50
MEX_Chig_S2_50
RSA_SRR5214706_50
ZNL_SRR10063240_34.5
ZNL_SRR10063242_27.2
ZNL_SRR10095341_30
Legend (Taxa):

B

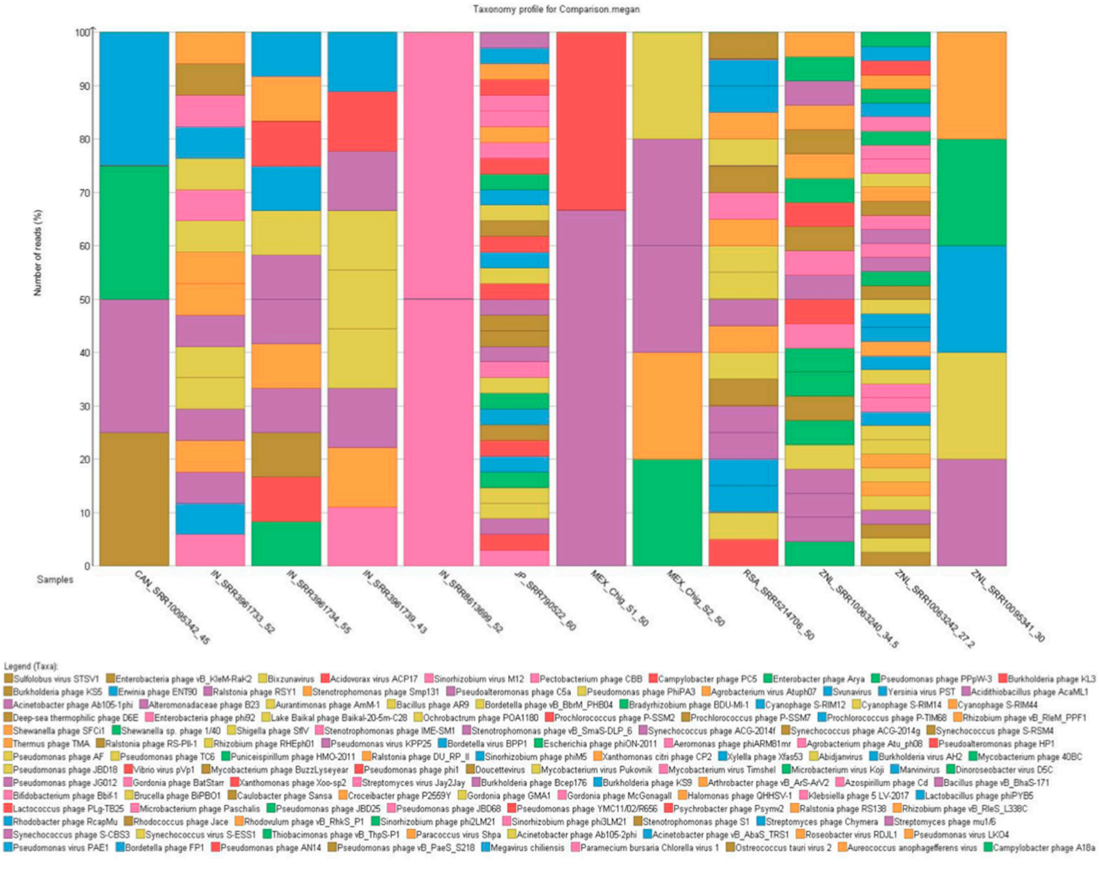
Taxonomy profile for Comparison.megan
Number of reads (%)
Samples
CAN_SRR10095342_45
IN_SRR3961733_52
IN_SRR3961734_55
IN_SRR3961739_43
IN_SRR8613699_52
JP_SRR790522_60
MEX_Chig_S1_50
MEX_Chig_S2_50
RSA_SRR5214706_50
ZNL_SRR10063240_34.5
ZNL_SRR10063242_27.2
ZNL_SRR10095341_30
Legend (Taxa):

Figure S7. Species of virus identification A) Vibrant and B) Virsorter.

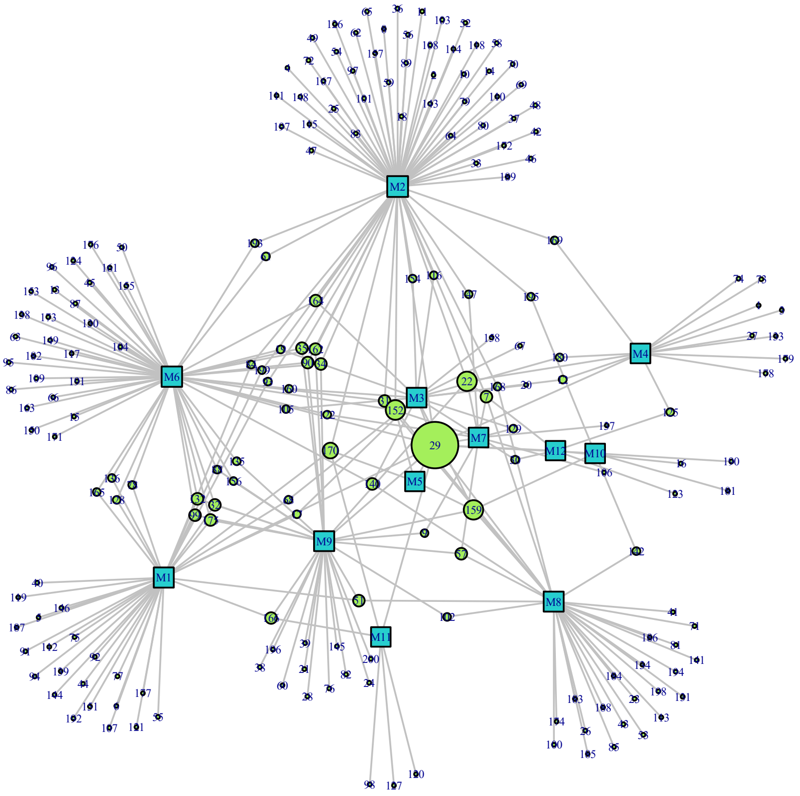

Figure S8. Virus network analysis. Metagenomes (M1) ZNL_SRR10063242_27.2, (M2) JP_SRR7905022_60, (M3) IN_SRR3961734_55, (M4) Mex_Chig_S1, (M5) ZNL_SRR10063241_30, (M6) ZNL_SRR10063240_34.5, (M7) IN_SRR3961739_43, (M8) RSA_SRR5214706_50, (M9) IN_SRR3961733_52, (M10), IN_SRR8613699_52, (M11) CAN_SRR10095342_45, (M11) Mex_Chig_S2. Virus species 1) *Bacillus virus* Bobb, 2) *Bacillus virus* Bcp1, 3) *Nitunavirus*, 4) *Siminovitchvirus* 5) *Enterobacteria phage* vB_KleM-RaK2, 6) *Bixzunavirus*, 7) *Acidovorax virus* ACP17, 8)*Sinorhizobium virus* M12, 9) *Pectobacterium phage* CBB, 10) *Campylobacter virus* Los1, 11) *Campylobacter phage* PC5, 12) *Enterobacter phage* Arya, 13) *Pseudomonas phage* PPpW-3, 14) *Erwinia phage* vB_EamM_Parshik, 15) *Acinetobacter virus* ME3, 16) *Acinetobacter virus* LZ35, 17) *Burkholderia phage* KS5, 18) *Burkholderia phage* vB_BceM_AP3, 19) *Ralstonia phage* RSY1, 20) *Stenotrophomonas phage* Smp131, 21) *Pseudoalteromonas phage* C5a, 22) *Agrobacterium virus* Atuph07, 23) Svunavirus, 24) *Enterobacter virus* PG7, 25) unclassified *Tequatrovirus*, 26) *Yersinia virus* PST, 27) *Acinetobacter phage* Acj9, 28) *Morganella phage* vB_MmoM_MP1 29) *Acidithiobacillus phage* AcaML1, 30) *Acinetobacter phage* Ab105-1phi, 31) *Alteromonadaceae phage* B23, 32) *Aurantimonas phage* AmM-1, 33) *Bacillus phage* AR9, 34) *Bordetella phage* vB_BbrM_PHB04, 35) *Bradyrhizobium phage* BDU-MI-1, 36) *Caulobacter phage* Cr30, 37) *Cyanophage* S-RIM12, 38) *Cyanophage* S-RIM14, 39) *Cyanophage* S-RIM32, 40) *Cyanophage* S-RIM44, 41) Deep-sea thermophilic phage D6E,

42) *Faecalibacterium phage* FP_Mushu, 43) Lake Baikal phage Baikal-20-5m-C28, 44) *Ochrobactrum phage* POA1180, 45) *Prochlorococcus phage* P-HM1, 46) *Prochlorococcus phage* P-SSM2, 47) *Prochlorococcus phage* P-SSM7, 48) *Prochlorococcus phage* P-TIM68, 49) *Pseudomonas phage* Lu11, 50) *Pseudomonas phage* PaBG, 51) *Rhizobium phage* vB_RleM_PPF1, 52) *Salicola phage* SCTP-2, 53) *Shewanella phage* SFCi1, 54) *Shewanella* sp. phage 1/40, 55) *Shigella phage* SfIV, 56) *Stenotrophomonas phage* IME-SM1, 57) *Stenotrophomonas phage* vB_SmaS-DLP_6, 58) *Synechococcus phage* ACG-2014f, 59) *Synechococcus phage* ACG-2014g, 60) *Thermus phage* TMA, 61) *Xanthomonas phage* XacN1, 62) *Yersinia phage* phiR1-37, 63) *Edwardsiella virus* MSW3, 64) *Enterobacteria phage* J8-65, 65) *Pseudomonas phage* vB_PaeP_PAO1_Ab05, 66) *Ralstonia phage* RS-PII-1, 67) *Ralstonia phage* RsoP1EGY, 68) *Rhizobium phage* RHEph01, 69) *Erwinia phage* vB_EamP-S2, 70) *Escherichia virus* Pollock, 71) *Erwinia virus* Frozen, 72) *Pseudomonas virus* KPP25, 73) *Escherichia phage* APC_JM3.2, 74) *Bordetella virus* BPP1, 75) *Enterobacter phage* Tyrion, 76) *Aeromonas phage* phiARM81mr, 77) *Agrobacterium phage* Atu_ph08, 78) *Burkholderia phage* vB_BmuP_KL4, 79) *Cellulophaga phage* phi46:3, 80) *Cellulophaga phage* phi14:2, 81) *Delftia phage* RG-2014, 82) *Pseudoalteromonas phage* HP1, 83) *Pseudomonas phage* AF, 84) *Pseudomonas phage* TC6, 85) *Pseudomonas phage* ZC08, 86) *Puniceispirillum phage* HMO-2011, 87) *Ralstonia phage* DU_RP_II, 88) *Ralstonia phage* RSK1, 89) *Sinorhizobium phage* PBC5, 90) *Sinorhizobium phage* phiM5, 91) *Xanthomonas citri phage* CP2, 92) *Xylella phage* Xfas53, 93) *Burkholderia virus* AH2, 94) *Streptomyces phage* Maneekul, 95) *Mycobacterium virus* Vincenzo, 96) *Mycobacterium virus* Godines, 97) *Mycobacterium phage* 40BC, 98) *Pseudomonas phage* JBD18, 99) *Gordonia virus* Bowser, 100) *Gordonia virus* Britbrat, 101) *Pseudomonas phage* MP42, 102) *Vibrio virus* pVp1, 103) *Rhodobacter virus* RcCronus, 104) *Stenotrophomonas virus* DLP5, 105) *Pseudomonas phage* phi1, 106) *Doucettevirus*, 107) *Mycobacterium virus* Pukovnik, 108) *Mycobacterium virus* Timshel, 109) *Mycobacterium phage* HINdeR, 110) *Escherichia phage* ST2, 111) *Microbacterium virus* Koji, 112) unclassified *Laroyevirus*, 113) *Vibrio virus* MAR10, 114) *Marvinvirus*, 115) *Dinoroseobacter virus* D5C, 116) *Mycobacterium virus* Panchino, 117) *Pseudomonas virus* PaMx25, 118) *Pseudomonas phage* JG012, 119) *Gordonia virus* Zirinka, 120) *Gordonia phage* BatStarr, 121) *Pseudomonas phage* AAT-1l, 122) *Xanthomonas phage* Xoo-sp2, 123) unclassified Pbi1virus, 124) *Escherichia phage* YDC107_1, 125) *Streptomyces virus* Jay2Jay, 126) *Gordonia virus* OneUp, 127) *Burkholderia phage* Bcep176, 128) *Burkholderia phage* KS9, 129) unclassified *Timquatrovirus*, 130) *Arthrobacter phage* vB_ArS-ArV2, 131) *Azospirillum phage* Cd, 132) *Bacillus phage* vB_BhaS-171, 133) *Bifidobacterium phage* Bbif-1, 134) *Caulobacter phage* CcrColossus, 135) *Caulobacter phage* Sansa, 136) *Cellulophaga phage* phi19:1, 137) *Corynebacterium phage* LGCM-V4, 138) *Croceibacter phage* P2559Y, 139) *Erysipelothrix phage* phi1605, 140) *Geobacillus virus* E2, 141) *Geobacillus virus* E3, 142) *Gordonia phage* Confidence, 143) *Gordonia phage* GMA1, 144) *Gordonia phage* GMA2, 145) *Gordonia phage* McGonagall, 146) *Halomonas phage* QHHSV-1, 147) *Klebsiella phage* 5 LV-2017, 148) *Lactobacillus phage* PLE3, 149) *Lactococcus phage* PLg-TB25, 150) *Microbacterium phage* Paschalis, 151) *Pseudomonas phage* JBD25, 153) *Pseudomonas phage* JBD44, 154) *Pseudomonas phage* JBD68, 155) *Pseudomonas phage* phiPSA1, 156) *Pseudomonas phage* PS-1, 157) *Pseudomonas phage* YMC11/02/R656, 158) *Psychrobacter phage* Psymv2, 159) *Ralstonia phage* RS138, 160) *Rhizobium phage* 16-3, 161) *Rhizobium phage* vB_RleS_L338C, 161) *Rhodobacter phage* RcapMu, 162) *Rhodococcus phage* Jace, 163) *Rhodovulum phage* vB_RhkS_P1, 164) *Sinorhizobium phage* phi2LM21, 165) Sinorhizobium phage phi3LM21, 166) *Sinorhizobium phage* phiLM21, 167) *Stenotrophomonas phage* S1, 168) *Streptococcus phage* phiZJ20091101-1, 169) *Streptomyces phage* Chymera, 170) *Streptomyces phage* Ibantik, 171) *Streptomyces phage* mu1/6, 172) *Synechococcus phage* S-CBS3, 173) *Synechococcus virus* S-ESS1, 174) *Thiobacimonas phage* vB_ThpS-P1, 175) *Paenibacillus phage* Dragolir, 176) *Paracoccus virus* Shpa, 177) *Acinetobacter virus* R3177, 178) *Acinetobacter phage* Ab105-2phi, 179) *Acinetobacter phage* vB_AbaS_TRS1, 180) *Gordonia virus* Vivi2, 181) *Roseobacter virus* RDJL1, 182) *Pseudomonas virus* LKO4, 183) *Pseudomonas virus* MP1412, 184) *Pseudomonas virus* PAE1, 185) *Bordetella phage* FP1, 186) *Pseudomonas phage* AN14, 187) *Pseudomonas phage* vB_PaeS_S218, 188) *Marinomonas phage* YY, 189) *Cyprinid herpesvirus* 2, 190) *Ictalurid herpesvirus* 1, 191) *Megavirus chiliensis*, 192) *Moumouvirus*, 193) *Tupanvirus* soda lake, 194) *Paramecium bursaria Chlorella virus* 1, 195) *Ostreococcus tauri virus* 2, 196) Yellowstone lake *phycodnavirus* 1, 197) Yellowstone lake *phycodnavirus* 2, 198) *Campylobacter phage* A18a, 199) *Synechococcus phage* S.

Table S1. Metagenome data considered for the analysis.

Strategy	Accession number	Number of run	Temp ºC	pH	Coordinates	Country	Year
wgs	SRX1982251	SRR3961734	55	7.8	22.65N 78.36E	India	2014
wgs	SRX1982250	SRR3961733	52	7.8	22.65N 78.36E	India	2014
wgs	SRX1982257	SRR3961739	43	7.8	22.65 N 78.36 E	India	2014
wgs	SRX6797119	SRR10063240	34.5	6.9	29.263502 S 177.920697 W	New Zeland	2016
wgs	SRX6797117	SRR10063242	27.2	5.43	29.262380 S 177.919075 W	New Zeland	2016
wgs	SRX6797118	SRR10063241	30	5.9	29.262380 S 177.920850 W	New Zeland	2016
wgs	SRX4741380	SRR7905022	60	5.4	34.318 N 139.216 E	Japan	2016
wgs	SRX1499016	SRR3050168	50	7	19º34’ N 78º22’E	India	2016
wgs	SRX2525297	SRR5214706	60	5.7	NI	South Africa	2017
wgs	SRX5412735	SRR8613699	42 to 52	7	22°55'30" N 73°12'33" E	India	2019
wgs	SX6827591	SRR10095342	42	7	59.431N 126.1 W	Canada	2019
